# Supplementary material for: Uncertainty in non-CO2 greenhouse gas mitigation contributes to ambiguity in global climate policy feasibility
Source: Nat Commun. 2023 Jun 2;14:2949. doi: 10.1038/s41467-023-38577-4 (PMC10238505; doi:10.1038/s41467-023-38577-4)
Supplement: Supplementary file 1 — Supplementary Information [file 41467_2023_38577_MOESM1_ESM.pdf]

Supplementary information

S1 NCGG Marginal Abatement Cost curve data used by IAMs

Table S1.1: Representation of non-CO_2 mitigation in 9 IAMs – Status: March 2023. Based on a follow-up of a stock take questionnaire from ref.[1]. "Explicit technologies" implies that a model endogenously calculates its source-specific reduction potentials, based on a bottom-up representation of available technologies. Note: model-specific additional assumptions are listed under the table. For IMAGE and Remind, the first reference is assumed for the short-term, the second reference for the long-term. The POLES fuel combustion CH_4 MAC is implemented for the residential and service sector. NM = No mitigation, N.a. = Category is not represented.

		AIM/CGE	*DNE21+*	*ENV-Linkages*	*GCAM*	*IMAGE*	*MESSAGE-GLOBIOM*	*POLES*	*REMIND-MAgPIE*	*WITCH-GLOBIOM*
Agriculture	**Wetland rice**	5	6	6	1	This study	3	3	This study	3
	**Livestock**	5	6	6	1	This study	6	3	This study	6
	**Fertilizer**	5	6	6	1	This study	6	3	This study	6
Fugitive emissions	**Coal / Oil / Natural gas**	5	6	6	1	2, This study	4	9	4, 5	4
Waste	**Landfills & Sewage**	5	6	6	1	1, This study	7	9	4, 5	4
Industrial processes	**Steel & Chemical**	NM	8	4	NM	NM	NM	N.a.	NM	NM
	**Bulk**	NM	8	6	NM	NM	NM	N.a.	NM	NM
Fuel combustion	**Residential sector**	NM	8	6	NM	NM	NM	9	NM	NM
	**Bunkers**	NM	N.a.	N.a.	NM	NM	NM	N.a.	NM	NM

Additional assumptions and modifications:

AIM/CGE: MAC data is fed into the AIM-Enduse model and fitted with an exponential curve (based on ref. [2]), leading to higher sectoral MRPs. **DNE21+ & ENV-Linkages:** Reduction potentials US-EPA (2019)[3] in 2020 below 1000$/tCO2 are extrapolated using Hyman et al.(2002)[4] exponential curve. **MESSAGE-GLOBIOM:** Agriculture: Technical potential in US-EPA (2006)[5] is corrected to reach lower economic potential. The model can also steer towards more GHG efficient livestock production systems[6] leading to much higher net MRPs. In MESSAGE-GLOBIOM, technologies are explicitly represented. **POLES:** Extrapolation of mitigation potential of agricultural emission sources to 74% in 2100 (will be corrected in future model version). **WITCH-GLOBIOM:** After 2030, abatement costs are decreasing by 1% per year and the total abatement potential is increasing by 1% per year.

References from most recent to oldest:

This study (2023)
1) US-EPA, 2019[3]
2) GAINS model (2018)[7,8]
3) GLOBIOM model (2018)[9]
4) US-EPA, 2013[10]
5) Lucas et al, 2007[11]
6) US-EPA, 2006[5]
7) Rao & Riahi, 2006[12]
8) Hyman et al., 2002[4]
9) GECS, 2002[13]

S2 IMAGE 3.2

The IAM used for this study is IMAGE 3.2[14,15]. IMAGE 3.2 is suitable for large scale and long-term assessments of interactions between human development and the environment. It integrates a range of sectors, ecosystems and indicators. One of the advantages is that it does not average over larger areas, but it models processes by means of unique grid cells. This differentiates between local conditions.

Model components and structure

The components of the IMAGE model are shown here[1] (note that the structure applies to the last versions; 3.0 and 3.2). Arrows indicate interrelations between subsystems. The framework exists of two main systems, the human system and the earth system. Linkages exist in both directions. Inputs to the model are descriptions of future development of drivers of global environmental change like, among others, population, economic development, lifestyle parameters, technological change, and trade regimes, tariffs and barriers. Since the exact development of these drivers is uncertain, different input values are used to understand the possible impact of the range of future developments. The current situation is used as a starting point and future projections are made until 2050 and 2100.

Scenarios

IMAGE 3.2 is used to develop and assess future global scenarios. These are, among others, Shared Socio-economic Pathways (SSPs)[15]. There are 5 SSPs, the scenarios SSP1 to SSP5. In this study, the scenarios SSP1, SSP2 and SSP3 will be used. SSP1 includes a world of sustainability-focused growth and equality. SSP2 covers the middle of the road estimates where social, economic and technological trends do not shift markedly from historical patterns. In the SSP3 scenario, a fragmented world of 'resurgent nationalism' is assumed. This is a world where concerns about competitiveness, security and regional conflicts push countries to increase their focus on regional problems instead of global problems. These different scenarios can be run for different climate targets[16].

Output

An IMAGE run produces data starting from 1970 to a final 'base year' that can be adjusted. The runs from 1971 to 2005 are used to test the model against key historical trends. The model has a long list of outputs, including

- energy use, conversion and supply
- agricultural production, land cover and land-use
- nutrient cycles in natural and agricultural systems
- emissions to air and surface water
- carbon stocks in biomass pools, soils, atmosphere and oceans
- atmospheric emissions of greenhouse gases and air pollutants
- concentration of greenhouse gases in the atmosphere and radiative forcing
- changes in temperature and precipitation; sea-level rise
- water use for irrigation

---

[1] https://models.pbl.nl/image/index.php/Framework_overview

Spatial resolution / Regions

There are 26 regions in IMAGE to capture spatial and multi-scale differences, see the country distribution here.[2] These regions are different in terms of location-specific biophysical conditions and the level of human development. Besides that, each region has differences in policy interventions, governance structures, cultures, and political factors.

IMAGE provides a relatively high level of detail on land-based processes, also in temporal and spatial resolution. Land use, land cover, and associated biophysical processes (such as water, carbon and nutrient cycles, and derived indicators for biodiversity loss and flood risks) are treated at the grid level to capture local dynamics. The grid size has been reduced to 5 x 5 arcminutes (corresponding to 10 x 10 km at the equator). Operating within global boundaries, the regional approach provides insight to identify where specific problems manifest, where the driving factors are concentrated, and how changes in some regions influence other regions.

[2] https://models.pbl.nl/image/index.php/Region_classification_map

S3 Emission coverage

Figure S3.1a: Anthropogenic non-CO_2 GHG emissions by emission source in 2020. The share is based on total CO_2eq (based on AR4 100-yr Global Warming Potential (GWP). Source: IMAGE SSP2[14,17]. No MAC curves have been developed for the light-shaded CH_4 and N_2O sources. Minor sources not shown in the figure, but are provided in Table S3.1: Agricultural Waste Burning (N_2O, CH_4), Biological N-fixation (N_2O), Biomass Burning(N_2O), and Industry (N_2O, CH_4). NCGGs are sorted clockwise from large to small.

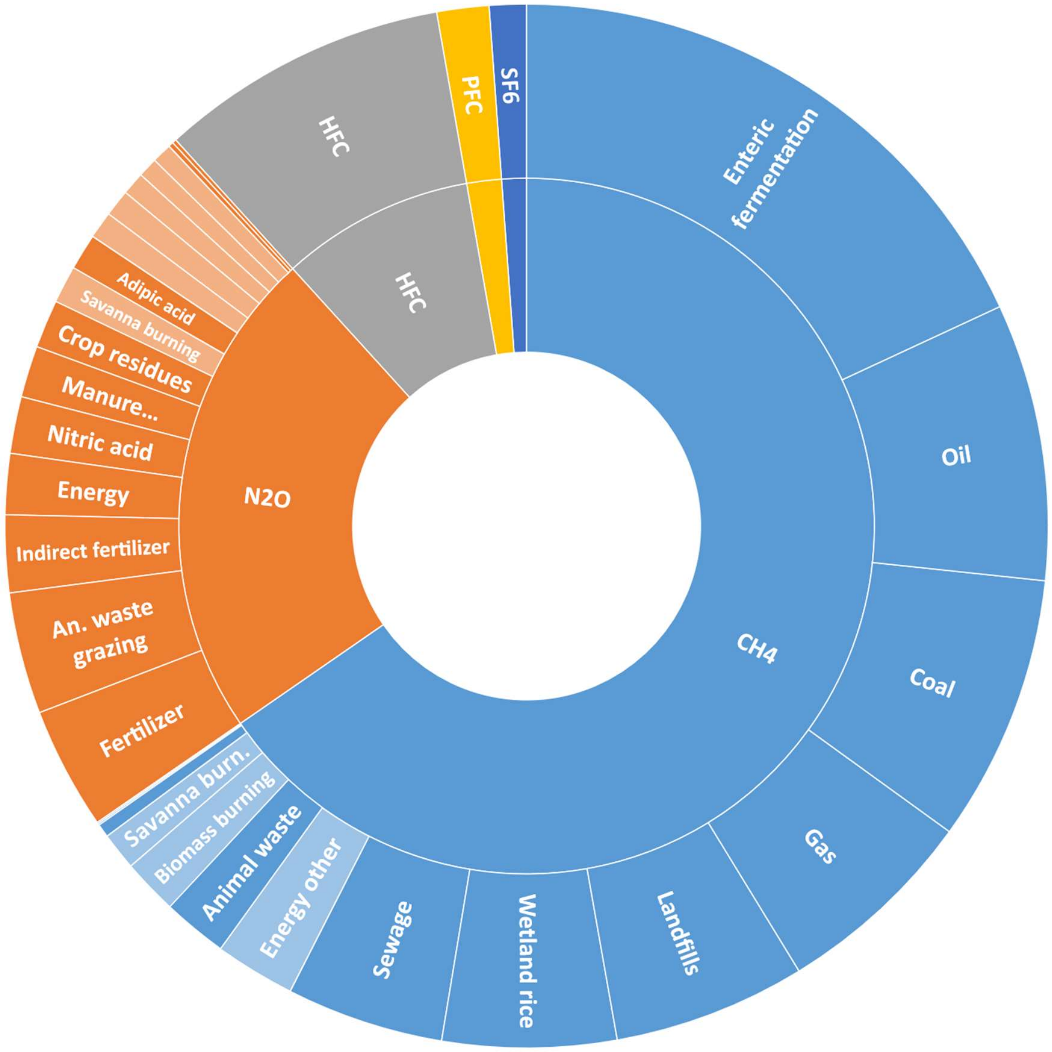

Figure S3.1b: Anthropogenic non-CO_2 GHG emissions by emission source in 2100. The share is based on total CO_2eq (based on AR4 100-yr Global Warming Potential (GWP). Source: IMAGE SSP2[14,17]. No MAC curves have been developed for the light-shaded CH_4 and N_2O sources. Minor sources not shown in the figure, but are provided in Table S3.1: Agricultural Waste Burning (N_2O, CH_4), Biological N-fixation (N_2O), Biomass Burning(N_2O), and Industry (N_2O, CH_4). NCGGs are sorted clockwise from large to small.

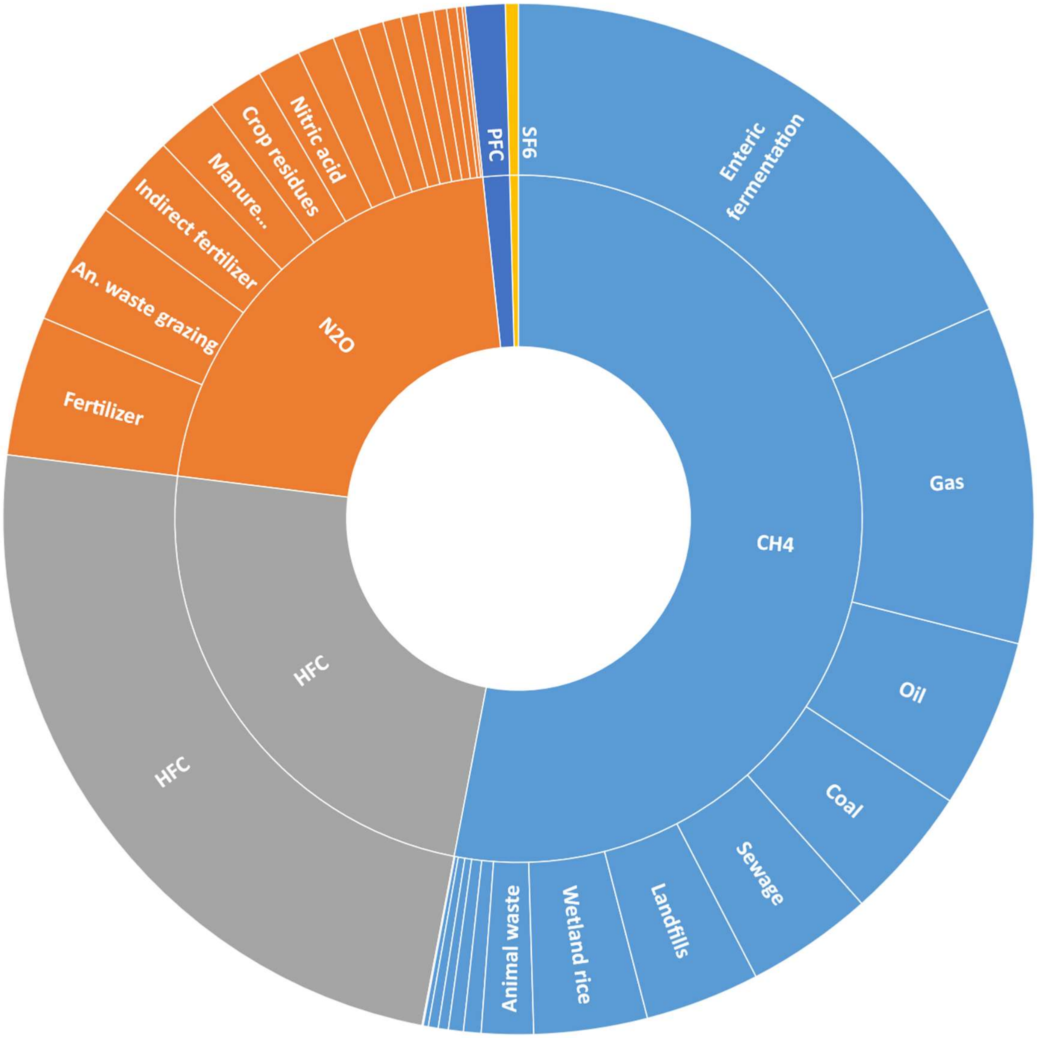

Table S3.1: Anthropogenic non-CO_2 GHG emissions, share of total CO_2eq in 2020 and 2100 in the IMAGE SSP2 scenario[14,17] with calibrated CH_4 emissions from fossil energy sources based on GAINS[7]. Data is provided in absolute terms i.e., total CO_2eq (based on AR4 100-yr Global Warming Potential (GWP)) and relative terms, i.e., share of total CO_2eq. Grey-shaded cells indicate sources without a MAC curve.

GHG	Source	2015		2100	
		Emissions (Mt CO_2eq/yr)	*% of total non-CO_2*	*Emissions (Mt CO_2eq/yr)*	*% of total non-CO_2*
**CH_4**	Enteric fermentation	2745	20%	3727	17%
**CH_4**	Oil	1339	10%	1113	5%
**CH_4**	Coal	891	7%	2168	10%
**CH_4**	Natural gas	860	6%	1724	8%
**CH_4**	Landfills	819	6%	772	4%
**CH_4**	Wetland rice	800	6%	876	4%
**CH_4**	Sewage	674	5%	843	4%
**CH_4**	Energy other	332	2%	142	1%
**CH_4**	Animal waste	300	2%	338	2%
**CH_4**	Biomass burning	233	2%	54	0%
**CH_4**	Savannah burning	158	1%	68	0%
**CH_4**	Agricultural waste burning	46	0%	51	0%
**CH_4**	Industry	7	0%	22	0%
**CH_4 Total**		**9203**	**67%**	**11900**	**55%**
**N_2O**	Manure grazing	591	4%	653	3%
**N_2O**	Fertilizer	570	4%	883	4%
**N_2O**	Indirect fertilizer	362	3%	513	2%
**N_2O**	Nitric acid	277	2%	385	2%
**N_2O**	Energy	255	2%	422	2%
**N_2O**	Manure application	250	2%	347	2%
**N_2O**	Crop residues	184	1%	374	2%
**N_2O**	Adipic acid	180	1%	178	1%
**N_2O**	Savannah burning	157	1%	68	0%
**N_2O**	Manure stables	116	1%	142	1%
**N_2O**	Domestic sewage	88	1%	103	0%
**N_2O**	Biological N-fixation	87	1%	135	1%
**N_2O**	Biomass burning	83	1%	19	0%
**N_2O**	Chemicals	19	0%	53	0%
**N_2O**	Agricultural waste burning.	14	0%	15	0%
**N_2O Total**		**3234**	**24%**	**4291**	**20%**
**HFC**	HFC	882	6%	5222	24%
**PFC**	PFC	191	1%	89	0%
**SF_6**	SF6	159	1%	265	1%
**Total**		13670		21767	
**Total % covered by MAC curve:**			**92%**		**96%**

S4 Approach for constructing the MAC curves (based on Harmsen et al., 2019)

S4.1 System boundaries

The MAC curves and scenario assessment in this study are based on the emission source categories of the IMAGE 3.2 model[14,15] as applied in Harmsen et al 2019[18], representing all anthropogenic non-CO_2 greenhouse gases. See supplement S3 for an overview of these emissions in 2020. The MAC curves provided in this study cover 90% of the present-day non-CO_2 GHG emissions and 95% of the projected emissions in 2100. The missing sources are either small or are indirectly included in the scenarios. Emission sources not covered in the study (light-shaded in S3) are nitrous oxide (N_2O) and methane (CH_4) emissions from: 1) Land clearing for agricultural extension (biomass burning and savannah burning) since this is driven by activities, rather than emission-intensities 2) Combustion (traditional biomass use for heating and cooking and transportation fuels), 3) Agricultural waste burning, and 4) Industry emissions (mainly iron and steel production and the chemical sector). The MAC curves developed in this study represent potential emission reductions under CO_2 equivalent prices up to 4000 $(2010)/tCeq (or 1091 $(2010)/t$CO_2$eq), the maximum price that is applied in the IMAGE IAM framework. Emissions and emission reductions are calculated for the 26 global IMAGE regions (see supplement S2). Regional differences in present day emission intensities and activities are fully represented in the scenario assessment. Regional emissions in the base year (2015 to 2020, depending on the source) are calibrated with data from several detailed databases covering different emissions sources; CEDS[19], GAINS[7], EDGAR 4.2.3[20,21].

S4.2 Construction of the MACs

The construction of the MAC curves is based on the method by ref.[18]. This section therefore also includes literal parts of the methods from that paper. The MACs are built up from individual source-specific measures and assumptions on long-term developments. This fully bottom-up approach has been applied to the agricultural MACs. For the non-agricultural MACs, elements of the bottom-up approach have been applied where possible. Figure S4.1 gives a schematical overview of the approach for one fictional emission source with two reduction measures, A and B. Equations S1 and S2 provide the underlying calculation.

Figure S4.1: General method for construction of the MAC curves[18]. Left graphs: differences between theoretical (A' and B') and actual (A and B) reduction potentials and costs, in relation to MAC components (TA: Technical applicability, RE: Reduction efficiency, IP: Implementation potential, OVcorr: Correction for overlap between measures, only influences measure B, RP: Reduction potential). Right graph: MAC curve made up of two measures (TP: Technological progress. Bcorr: Correction for emission reductions in the baseline scenario, MRP: Maximum reduction potential of measures combined). ω represents the cost increase (old minus new) for measure B, due to diminishing returns.

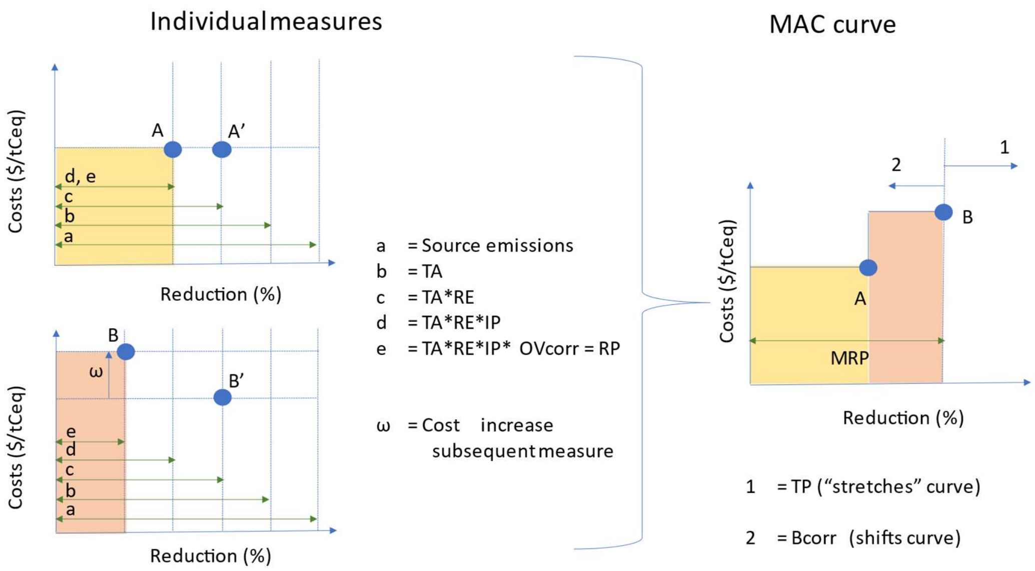

The relative reduction potential (RP) (in %) of each individual mitigation measure in year t and region r is determined by eq. S1. The maximum reduction potential (MRP) (in %) is the maximum relative abatement compared to baseline source emissions when all source-specific measures are implemented (eq. S2).

$$RP_{(t,r)} = RE * TA_{(r)} * OVcorr_{(t,r)} * IP_{(t)} \quad (S1)$$

$$MRP_{(t,r)} = (RP_{1\,(t,r)} + RP_{2\,(t,r)} + RP_{3\,(t,r)} \dots + RPx_{(t,r)}) * TP_{(t)} - Bcorr_{(t,r)} \quad (S2)$$

With (all in %): TA: Technical applicability, This is the part of the baseline that can technically be covered by the measure. This is often 100%, but can be lower e.g., if only a sub-process is targeted or if regional climatic circumstances are unsuitable. RE: Reduction efficiency, i.e., relative reduction in case a measure can be applied, generally based on multiple case studies. IP: Implementation potential, represents (the lack of) non-technical barriers. This is assumed to increase over time due to increased technology diffusion and policy acceptance. OVcorr: Correction factor for overlap between measures that target the same emissions. It is assumed that the least costly measures are implemented first. If a subsequent measure is applied, it has a diminished benefit due to lower remaining emissions. Note that this correction increases in time (i.e., a lower value for OVcorr) as IP increases (see detailed description below) TP: Technological progress, implemented in time as the increase of the reduction potential in time, as a result of new or improved technologies. This is the only factor that is larger than 100% (see detailed description below) Bcorr: Correction factor for regional emission reductions that already occur in the baseline scenario, e.g., due to zero or negative cost measures, such as the use of

fugitive CH_4 emissions as an energy source, or non-climate policy reductions, such as from air quality measures.

Regional differences in mitigation potential are included where these are known. These differences are reflected in the parameters: technical applicability, reduction efficiency, and costs. Partly, these are due to socio-economic circumstances (e.g., different levels of advancements in farming techniques) that can have short-term implications on mitigation potentials. However, in cases of similar biophysical circumstances across regions, we assume convergence in mitigation potentials (i.e., minimum emission intensities) in the long term and at maximum carbon prices. Where differences in mitigation potentials are known to be caused by biophysical regional differences, such as regional temperature, precipitation, geography, etc., this has been included in the form of quantitative constraints on the components underlying the MACs. In this study, we differentiated between regions that have high, medium and low technical applicability (see supplement S7) for enteric fermentation and CH_4 manure, based on the GAINS model Global CH_4 mitigation potentials for livestock in 2030 and 2050[22]. Regional differences in reduction efficiency are incorporated in the measure 'anaerobic digestion', which has different known impacts in warm and cold environments. Regional differences in costs are incorporated where available (see tables S7.2 and S7.3). It is known that costs can be different across regions, for instance due to differences in labor costs, costs of capital (with the last two factors typically being negatively correlated), energy and resource requirements and climate-related durability. Unfortunately, in most cases, very little direct information on regional cost differences can be found in literature, in which case we assumed an aggregated global estimate.

Correction for overlap

Mitigation measures can "overlap" when they are aimed at reducing the same emissions. This depends on two factors: 1) the share of other source-specific measures already in place and 2) The overlap between targeted emissions by two different measures. Regarding 1: If a subsequent measure is applied next to one or more measures already in place, it can have a diminished benefit since less emissions remain (If measure y is aimed at reducing the same baseline emissions as measure x that is already implemented, the $OVcorr_y = 1\text{-}RP_x$). Note that this correction increases as implementation increases. Regarding 2: The overlap under 1 is less when measures target (partly) different baseline emissions. Smith, et al. [23] estimated how much measures differ in terms of targeting source-specific baseline emissions. If this value is "Diff", then $OVcorr_y = 1\text{-}(RP_x * (1\text{-}Diff_{x\text{-}y}))$. A value of 100% represents full difference/no overlap, 70%/50%/20% are used for low/medium/high overlap, respectively. The first measure that is implemented has no overlap and therefore, Diff = 100%. For each subsequent measure, the correction for overlap is the product of the overlap values with previously implemented measures.

Technological progress

It can be expected that the future MRP values are higher than the MRP values derived from the MACs (i.e., "MRP_{MAC}" in equations S3 and S4), with all the abatement measures from this assessment fully included, due to new and improved technologies. It can also be expected that this holds more in the far future. The exact effect is highly uncertain, with little to no available literature, so we use a very

aggregated (applying TP over the overall MAC, rather than individual sources) and conservative approach (with relatively low values). It is assumed that in 2050 and 2100, after implementing all measures included in the MAC the remaining emissions can be reduced by 10% and 20% more, respectively. So:

$$MRP_{2050} = 1 - (1 - MRP_{MAC}) * 90\% \qquad (S3)$$

$$MRP_{2100} = 1 - (1 - MRP_{MAC}) * 80\% \qquad (S4)$$

Between 2050 and 2100, the MRP values are linearly interpolated to determine an MRP for each year. It is conservatively assumed that the additional technology improvements occur at very high GHG prices: linearly increasing between 3000 \$/tCeq and 4000 \$/tCeq (or 818 \$/tCO_2eq and 1090 \$/tCO_2eq).

Marginal costs

The combination of measures with the highest estimated maximum reduction potential is used to construct MAC curves. It is assumed that the least costly measures are implemented first. When multiple measures are used, the mitigation cost increases, due to diminishing returns when measures overlap, with for any measure x:

$$\text{Cost new}_x = \text{Cost old}_x * 1/\text{OVcorr}_x \qquad (S4)$$

Note that ω in Figure S4.1 represents the cost increase (old minus new) for measure B. One consequence of this approach is that the more expensive measures also have a larger cost correction, since these are also the measures that are assumed to be implemented last. Another consequence is that marginal costs tend to be higher later in the century when implementation potentials and thus corrections for overlap are assumed to be higher.

Method by emission category

The default MAC curves for the non-agricultural sources are directly based on Harmsen et al. (2019)[18], with only a few, minor modifications to the default values for the maximum reduction potentials (MRPs), where this was justified by literature. These central estimates were complemented with optimistic and pessimistic MACs, with MRPs based on the literature study, which were used to scale the default MAC (see Supplement S5). Waste and industry MACs *(CH_4 from landfills/solid waste, CH_4 from sewage and wastewater, N_2O from adipic and nitric acid production, N_2O from transport, N_2O from Domestic sewage),* are based on data up to 2030 [3,10,11,13], but have added assumptions on the technological progress up to 2100, largely based on current best practices[18]. Fossil energy MACs *(CH_4 from coal, oil and gas production)* are based on a dataset from the GAINS model[7,8] with added long-term (MRP) assumptions on included promising technologies that are currently not in use on a large scale. The default F-gas MACs (*HFCs, PFCs and SF_6*) are directly copied from and described by Harmsen, et al. [18], with recent calibrations by Velders, et al. [21] and Schwarz, et al. [24].

S5 Input parameters pessimistic – default – optimistic MACs – non-Agriculture

The optimistic, default and pessimistic MACs for the non-agricultural sources have been developed by varying the maximum reduction potentials (MRPs) in 2050 and 2100 and scaling them in intermediate years. The default MACs are largely equal to those developed by ref. [18], with some small modifications (see table 5.1 below for the quantitative assumptions by source). Where known, estimates of current technical reduction potentials (based on projections by GAINS and US-EPA[3,22,25]) were used as a minimum value for the pessimistic MACs.

Table S5.1: MRP assumptions for the non-agricultural sources. Compared to MRP assumptions in Harmsen et al., (2019)

	Old MRP 2050 (%)	Old MRP 2100 (%)	New MRP 2050 Low-medium-high (%)	New MRP 2100 Low-medium-high (%)	Comments
**CH_4 Coal**	-	79	-	54-79-84	Low: global mean MRP [22], High = default [18] + 5%
**CH_4 Oil**	-	80	-	54-80-85	As CH_4 Coal
**CH_4 Gas**	-	90	-	82-90-95	As CH_4 Coal
**CH_4 landfill**	75	90	51-61-66	69-79-90	Low: global mean MRP [3], Default = Low + 5%, High [18]
**CH_4 wastewater**	62	90	36-46-66	49-59-90	As CH_4 landfill
**N_2O domestic sewage OECD**	50	65	30-50-67	41.1-64.8-84.9	RE range based on Sun, et al. [26]. TA*IP = default [18] ±5%
**N_2O domestic sewage non-OECD**	22.4	50.4	10.0-22.4-31.7	30.1-50.4-67	RE range based on Sun, et al. [26]. TA*IP = default [18] ±15%
**N_2O transport**	85	85	75-85-90	75-85-90	Low = default [18] – 10%, High = default [18] + 5%
**N_2O Aacid**	100	100	90-100-100	90-100-100	Low = default [18] – 10%
**N_2O Nacid**	94	94	84-94-96	84-94-96	Low = default [18] – 10%, High = default [18] + 2%
**HFCs**	96 to 100	96 to 100	Low: 90 Med/high:	Low: 92 to 95 Med/high: 96 to 100	Default[18], high[18] Low = minimum based on [3,22,27,28]
**PFCs**	80	90 to 95	Low: 54 to 77 Med/high:	Low: 75 to 80 Med/high: 90 to 95	As HFCs
**SF6**	80 to 90	90 to 100	Low: 60 to 70 Med/high:	Low: 75 to 80 Med/high: 90 to 100	As HFCs

The 2100 MRP for the pessimistic *CH_4 fossil fuel* MACs represents the global mean MRP in 2050 (as projected by GAINS[7,8]). The optimistic MRPs are the default MRPs with an added 5%points. Note: MRP values in the GAINS fossil fuel emissions are applied as the default long-term maximum values for regions that stay below this value in the short term. However, if regions can reach short-term reductions higher than the MRP, this is also assumed for the long term. This implies that the net global MRP can be higher than the default MRP, especially at low MRP values. For *CH_4 landfills and wastewater,* the MRP in 2050 of the pessimistic MAC was assumed to be the global average MRP in

2050 (as projected by the US EPA[3]). The pessimistic MRP in 2100 is based on the 2050 MAC with added assumptions on technological progress (2% increase in reduction potential per 5 years[18]). The optimistic MACs are based on the MACs from ref. [18], since these have been criticized to be overly optimistic. The default MACs are assumed to be 10% higher than the pessimistic MACs and to represent a middle-of-the-road case. For *N_2O domestic sewage,* the MACs from ref. [18] were built up from the parameters RE, TA and IP. RE values are based on a meta study by Sun, et al. [26]. Here, the pessimistic and optimistic MACs are based on the 95th and 5th percentile from the assessed measures in that study. The product of TA and IP is assumed to vary between ±15% (non-OECD) and ±5% (OECD). For both *N_2O transport, N_2O adipic acid and N_2O nitric acid*, the pessimistic maximum reduction potentials are assumed to be 10% points lower than ref.[18]. The optimistic MRPs are 5% points and 2% points higher, respectively.

The default and optimistic *F-gases* MACs are both directly based on ref. [18]. This default MAC assumes an already very high (>90%) mitigation potential for total F-gases, resulting in very low residual F-gas emissions in a mitigation case. The focus in determining the MRP range has therefore been on determining the minimum MRP, to be able to assess if a substantial increase in residual F-gas emissions in a mitigation scenario could be likely. The pessimistic F-gas MACs are based on additional GAINS and US-EPA insights on potential constraints on mitigation potentials[3,22,27]. For HFCs, it is unlikely that reductions are substantially lower than in the default case. A range of "non-in-kind" or other low-GWP alternatives are available as substitution for refrigeration and foams (e.g., cyclopentane in foams) as is avoidance (e.g., HFC-134a in polyurethane foam). The destruction efficiency for the HFC-23 waste (produced as a by-product of HCFC-22 production) is higher than 99.99%, and almost all the HCFC-22 production facilities in industrialized countries are well controlled[28]. In addition, HFC mitigation is strongly backed by ambitious climate policies (the Kigali Amendment to the Montreal Protocol since 2019, the Clean Development Mechanism of the Kyoto Protocol (with successful projects in China and India), national/regional regulations (e.g., in the USA, Japan and the EU)). The slightly lower MRPs in the pessimistic MACs (92-95% in 2100) correspond to a situation with imperfect policy enforcement and/or unidentified leakages. For PFCs and SF_6, a substantially lower MRP than the default is found to be more plausible than for HFCs. For both species, emissions from industrial processes are more uncertain, partly due to a lack of detailed activity data in semiconductor and electrical equipment production. SF_6 emission reductions from magnesium production are found to be more certain, due to the availability of zero- or low-GWP alternatives (e.g., Novec™ 612).

S6 Emission source-specific measures and assumptions

This supplementary section describes the emission sources, emission-reducing emissions and additional assumptions to construct the MAC curves. Note that large parts of the text are directly copied from [18], as the main structure and assumptions from that study are used here as well. Where relevant, this text is complemented with new insights from this study's literature assessment. Table S6.1 starts with an overview of the measures and assumptions per emission source, the main references and range in reduction efficiencies in the case of agriculture. The section continues with a description of the source-specific measures and assumptions. This description only includes CH_4 and N_2O sources, since the F-gas MACs are directly based on [18] (who also provide a description of the F-gas assumptions).

Table S6.1: Included measures, assumptions and references by emission source

	Measures	Long-term assumptions	Range in reduction efficiencies (%)	References reduction efficiencies	References datasets
**CH_4**					
CH_4 – Coal production	Pre-min degasification, Oxidation of ventilation air methane (VAM)	In 2050: Oxidation of lean (up to 0.5%) VAM feasible, abandoned mine CH_4 minimized<br>In 2100: Post-mining emission reduced by 50%[18]			GAINS[7,8]
CH_4 – oil production	Recovery and utilization of vented gas, reducing unintended leakage	In 2100: Small gas-to-liquid plants available for remote oil fields, monitoring of flares[18]			GAINS[7,8]
CH_4 – Natural gas production	Reduced leakage rates, installation PE and PVC networks	In 2100: LDAR (infrared cameras) to promptly find and close leakages			GAINS[7,8]
CH_4 – landfills/solid waste	Collection and flaring, LFG capture for energy use, Enhanced waste diversion (e.g., recycling, reuse)	2015-2100: Growth in reduction potential: 2% / 5 years, increased waste diversion and biological treatment[18]			US-EPA[3]
CH_4 – sewage and wastewater	Anaerobic digestion and CH_4 collection, wastewater treatment plants (wwtp) instead of latrines and disposal	2015-2100: Growth in reduction potential: 2% / 5 years[18]			US-EPA[3]
CH_4 enteric fermentation	Addition of nitrate to the feed		21-42	29-36	This study
	Genetic selection and breeding		8-31	37-41	This study
	Adding tannins as a food supplement		10-32	42-46	This study
	Grain processing		10-38	44,47-49	This study
	Improved health monitoring and illness prevention		4-20	39,50-52	This study
	Seaweed (Asparagopsis taxiformis)		12-99.5	53-58	This study
CH_4 - rice production	Rice straw mitigation		26.5-61	59-64	This study
	Direct seeding		16.6-47	59,63,65-67	This study
	Replacing urea with ammonium sulphate		14.18-42	59,63,68,69	This study
	Addition of phosphogypsum		28-86	59,63,70-73	This study
	Alternate flooding and drainage		18.8-79	45,59,63,64,74-91	This study

CH_4 manure	Manure acidification		61-98	[44,62,92-96]	This study
	Anaerobic digestion		25-75	[59,97-99]	This study
	Solid-liquid separation		46-81	[97,98]	This study
	Manure storage: duration		38-76	[100]	This study
	Housing systems and beddings		4-96	[29,44,101-105]	This study
	Manure storage covering		0-90	[29,44,94,106]	This study
**N_2O**					
N_2O fertilizer	Nitrification inhibitors		17-60	[10,29,107-117]	This study
	Improved land manure application		5-50	[114,118-122]	This study
	Irrigation practices		15-67	[123-126]	This study
	Biochar		14-38	[127-130]	This study
	Spreader maintenance		22-42	[59,131-133]	This study
	Improved agronomy practices		14-54	[122,134-139]	This study
	No tillage		25-48	[140-144]	This study
N_2O manure	Reduced dietary protein		0-52	[44,145-149]	This study
	Decreased manure storage time		35-35	[44]	This study
	Manure storage covering		30-75	[29,44]	This study
	Improved animal housing systems and bedding		9-88	[29,101,103,104]	This study
	Anaerobic digestion		34-75	[99,150,151]	This study
	Acidification		0-96	[152-157]	This study
N_2O transport	Low-N_2O catalytic converters for petrol cars	2015-2100: Growth in reduction potential: 2% / 5 years[18]			[11,13]
N_2O – Adipic acid production	Thermal decomposition (potentially combined with catalyst)	2015-2100: Growth in reduction potential: 2% / 5 years[18]			US-EPA [3]
N_2O – Nitric acid production	Catalic decomposition, thermal decomposition (potentially including reagent fuel)	2015-2100: Growth in reduction potential: 2% / 5 years[18]			US-EPA [3]
N_2O – domestic sewage	N-removal at wastewater treatment plants, N-enriched wastewater as an alternative to fertilizers	2015-2100: Growth in reduction potential: 2% / 5 years[18]			[11,18]
**F-gasses**					
HFCs – refrigeration	Substitution with low-/zero GWP substances, better sealed systems, recovery after use			[13,16]	[18,24]
HFCs – foams	Substitution with low-/zero GWP substances			[13,16]	[18,24]
HFCs – Production of HCFC-22 (HFC-23 by-product)	Thermal destruction			[13,16]	[11]
HFCs - other	Substitution with low-/zero GWP substances, better sealed systems, recovery after use				[18,24]
PFCs – primary	Switch to Point-Feed Prebake technology (PFPB)			[13,16]	[11]

Aluminium production					
PFCs – semiconductor manufacturing	Emission capture and (thermal) destruction			[13,16]	158
PFCs – other sources PFCs	Substitution with zero GWP substances			[13,16]	158
SF_6 – production of electrical equipment	Improved recovery and recycling, minimization of leakage (detection and repair), improved handling			[13,16]	11
SF_6 – Use and decommissioning of elec. Eq.	Improved recovery and recycling, minimization of leakage (detection and repair), improved handling			[13,16]	11
SF_6 – Magnesium production	Substitution with zero GWP substances			[13,16]	11
SF_6 – Other sources	Improved recovery and recycling, minimization of leakage (detection and repair), improved handling, substitution with zero GWP substances			[13,16]	11

Agriculture

CH_4 Rice

CH_4 emissions result from the anaerobic breakdown of organic matter in wetland rice paddies in the growing season. The CH_4 transports from the rice paddies into the air by diffusion and through the rice stems. The amount of methane emitted from the rice paddies depends on, among others, rice variety, soil type, climate, period that the paddies are flooded, amount and application of fertilizer, management[159].

The mitigation measures that have been used to construct the MAC curve are: 1) *Alternate flooding and drainage / alternate wetting and drying:* this measure reduces anaerobic conditions by flooding every time the surface water level naturally declines to 15 cm below the soil surface level[88]. 2) *Phosphogypsum:* Phosphogypsum is formed as a by-product of the production of fertilizer from phosphate rock. Addition of phosphogypsum to soil releases sulphate, which inhibits methanogenesis (production of methane by bacteria or other living organisms). However, it has a heavy metal content (specially Cd) and radionuclide content, and its use is therefore still limited[160-165]. 3) *Direct seeding:* DSR (direct seeded rice) refers to the process of growing a rice crop from seeds sown in the field rather than by growing seeds in a nursery and then planting them. This measure includes both wet and dry seeding, these cannot be used simultaneously. In wet seeded rice, the seeds are sown on or into puddled soil[65]. Dry direct seeding has potential to save both water and labor[166]. 4) *Replace urea with ammonium sulphate (AS):* replaces commonly used urea; sulphate inhibits methanogenesis[59]. 5) *Rice straw management.* Rice straw is a residual byproduct of rice production at harvest. Current practices in rice production leave a huge amount of wet straw on the field, which cannot be used as feed or for food. Open field burning of rice straw results in atmospheric pollution and reduced soil quality, therefore, it has a negative impact on the environment. An example of trace gases that are emitted by

burning of rice straw are GHGs including CO_2, CH_4, and N_2O, along with other trace gases that contribute to tropospheric ozone and the formation of Atmospheric Brown Cloud (ABC). The formation of ABC is a serious concern for human health[167-172]. Compost production is one way of effectively utilizing rice straw. Composting is a bio-oxidative process and the compost can be used as organic fertilizer[173,174]. Removing rice straw in flooded rice is considered another mitigation opportunity for CH_4 emissions[175]. Maximum emission reductions and yield (and soil organic carbon (SOC) deposition) may be best achieved by partial straw return/removal in most continuous rice systems[61].

CH_4 manure

Methane is produced from the decomposition of livestock manure under anaerobic conditions[176]. This mostly happens when manure is stored in large piles. The mitigation measures that have been used to construct the MAC curve are: 1) Decreased manure *storage time:* Reduced storage time through frequent land application to avoid the anaerobic conditions that create CH_4; this can also reduce N_2O emissions depending on application timing. 2) *Anaerobic digestion:* Application of anaerobic digester for either small-scale farm systems, or centralized plants in intensive agricultural areas. The biogas generated from anaerobic digestion is used to produce heat or both heat and electricity. 3) *Storage covering:* Covering manure storages with permeable or impermeable covers is an effective mitigation practice. However, with an impermeable cover the CH_4 captured under the cover is burned using a flare system or engine-generator to produce electricity; otherwise, the captured CH_4 would build pressure inside the storage creating an explosion hazard and/or escape through leaks and cover ruptures. *4) Manure acidification:* The pH range in manure can impact the growth and activity of different microbial groups. Most methanogens have an optimum pH of around 7[177-180]. By acidification of the slurry, methanogenic activity may be reduced. 5) *Housing systems and beddings:* Concrete slatted floors with drainage/flush systems result in fewer CH4 and N2O emissions than solid floors with hay or other bedding. 6) *Solid liquid separation*: Manure solids can be mechanically separated from manure liquids. The advantages are reduced storage capacity, increased flexibility of manure management and reduced CH_4 emissions[181,182].

N_2O manure

N_2O is generated during the composting of manure by ammonia (NH_3)-oxidizing bacteria. The mitigation measures that have been used to construct the MAC curve are: 1) *Decreased manure storage time:* Reduced storage time through frequent land application to avoid the anaerobic conditions that create CH_4; this can also reduce N_2O emissions depending on application timing. 2) *Anaerobic digestion:* Application of anaerobic digester for either small-scale farm systems, or centralized plants in intensive agricultural areas. The biogas generated from anaerobic digestion is used to produce heat or both heat and electricity. 3) *Storage covering:* Covering manure storage with permeable or impermeable covers is an effective mitigation practice. However, with an impermeable cover, the CH_4 captured under the cover is burned using a flare system or engine-generator to produce electricity; otherwise, the captured CH_4 would build pressure inside the storage creating an explosion hazard and/or escape through leaks and cover ruptures. *4) Manure acidification:* The pH range in manure can impact the growth and activity of different microbial groups. Most methanogens have an optimum pH of around 7[177-180]. By acidification of the slurry, NH_3-oxidizing activity may be reduced. 5) *Housing systems and beddings:* Concrete slatted floors with drainage/flush systems result in fewer CH_4 and N_2O emissions than solid floors with hay or other bedding. 6) *Reduced dietary protein:* this measure

aims at reducing animal protein intake beyond what they require. Studies with pigs, poultry, and cattle have shown reduced NH_3 and potentially N_2O emissions from manure.

N_2O fertilizer

Excess application of nitrogen-based fertilizer stimulates microbes in the soil to convert nitrogen (N) to N_2O. Emission reductions can be achieved by minimizing fertilizer overuse and altered microbial conditions. The mitigation measures that have been used to construct the MAC curve are: 1) *No tillage*: tillage increases greenhouse gas fluxes due to enhanced decomposition of soil organic carbon[183]. 2) *Improved land manure*: this includes reducing inorganic N application with allowance for manure/residual N, improved timing of slurry and manure application, separating slurry/manure applications from fertilizer applications by several days, applying manure to dry rather than wet areas, applying solid rather than liquid manure / applying liquid and solid manure separately. 3) *Improved agronomy:* The goal is to increase crop intensity and reduce N_2O emissions by adopting systems less reliant on inputs (nutrients, pesticides), plant varieties with improved N-use efficiency, and use of rotations with legume crops, use of catch or cover crops. Cover crops, for example, have the capacity to scavenge surplus N trough biological nitrogen fixation. As a result, the fertilizer demand is reduced[137]. 4) *Spreader maintenance:* avoiding over-application and under-application, among others using variable rate technology. 5) *Nitrification inhibitors:* Nitrification inhibitors such as DCD and Nimin reduces N_2O emission by slowing the conversion of ammonium to nitrate. 6) *Biochar:* applying biochar to soils may affect conditions that control nitrification and denitrification. This measure is only used in the optimistic MACs because of limited long-term case studies. 7) *irrigation practices (drip irrigation):* Drip irrigation is a water saving irrigation method. Drip irrigation reduces soil erosion, salinization, and agricultural runoff.

CH_4 Enteric fermentation

The mitigation measures that have been used to construct the MAC curve are the following (taking into account overlap between measures and aiming for the highest MRP): 1) *Improved health monitoring and illness prevention*: Controlling or eradicating endemic livestock diseases. 2) *Addition of nitrate to the feed*: The addition of electron receptors such as nitrates (NO_3) may reduce CH_4 emission and increase productivity by acting as an H_2 sink and adding ammonia-based nitrogen to the rumen. (Addition: 17-23 g / kg DM) max 70% TA because the nitrate content in US and EU is already quite high. Adding nitrate is also limited to the time of lactation (about 10 months per year). The technical applicability of adding nitrate is limited to a maximum of 70% because the nitrate content in the food is already quite high in the US and in Europe. 3) *Genetic selection and breeding*: Selection of low CH_4 generation and higher feed efficiency per unit of milk produced. 4) *Adding tannins as a food supplement*: Plant extracts such as tannins or saponins are very effective in reducing rumen CH_4 emissions. 5) Grain processing: Improving starch digestibility of grain through mechanical processing such as steam flaking instead of dry rolling may reduce CH_4 emission by 10%. This also improves productivity. 6) *Seaweed (Asparagopsis Taxiformis) as a feed additive*: This seaweed contains high concentrations of bromoform, a substance that counteracts the formation of methane in the cow's rumen. (Level of inclusion: 0.5%). This measure is only used in the optimistic MACs, since it is questionable if large-scale production of seaweed would be possible and unclear what the long-term effects are in ruminants (in terms of efficacy and health)[58] .

Fossil energy

CH_4 Coal production

Emission reduction measures exist for the underground mining of hard coal, the largest source of CH_4 emissions in coal production. Emissions from surface mining of hard coal and lignite currently cannot be mitigated, but are also much lower per ton of coal (and +/- 15% of total coal CH_4 emissions) owing to the low pressure and coal rank[184]. Ventilation air methane (VAM, with a low concentration of 0.1-0.8% CH_4) during underground mining operation constitutes the main emission source (50-60% of total coal CH_4 emissions), while the rest of the emissions come from pre-mining activities, post-mining activities and abandoned mines (sources of comparable size)[8,185]. The mitigation potential for pre-mining degasification is assessed at 90%, which has been seen as possible in the US and as such applied in the GAINS dataset. VAM is seen as difficult and uneconomic to combust and unlike the other sources, it is not a safety risk, so therefore less attractive to mitigate[185]. Since no future technological improvements are assumed in the GAINS dataset, it is assumed that only non-lean VAM (> 0.3% concentration, 66% of all VAM) can be reduced. However, in a recent study, it was found that lower temperature catalytic thermal oxidation of methane led to a 100% removal of VAM CH_4, which was maintained for 2 years[185]. Also, other studies describe high reduction efficiencies (90%-100%) in controlled experiments[186-189]. The assumption in this study is therefore that VAM can be fully reduced by 2050. Similarly, CH4 from abandoned mines is assumed to be fully abatable in 2050 in this study. Abandoned underground mines can liberate CH_4 at a low, but near-steady rate over an extended period. If the mine is flooded, this can be reduced to a few years. In addition, recovery or oxidation of CH_4 can in principle also be applied[184]. The GAINS dataset does not include mitigation options for post-mining CH_4 emissions (i.e., further processing of coal). Although no known literature exists regarding this source, we assume that in 2100, due to technological advances it is likely that 50% can be reduced by moving some of these activities indoors and applying existing CH_4 removal technologies there.

CH_4 Oil and natural gas production and distribution

CH_4 emissions in the oil and natural gas industry partly originate from unintended leakage from pipelines, wells and facilities, and partly from intended safety induced venting and flaring during maintenance and (oil) drilling. For oil production, emission reduction measures are available that have proven to bring down emissions considerably (leading to a projected 80% in 2050): Recovery and utilization of vented gas and reduction of unintended leakage from wells and temporary storage of captured CH_4[8,25,190]. These measures are often cost saving or are otherwise relatively economical (0-300 $(2010)/tCeq). In addition to these measures included in the GAINS dataset, it is assumed that in 2100, small gas-to-liquid plants become available for remote oil fields. Such a development would make it economically sound to recover, liquefy and market as much as possible of the associated petroleum gas, instead of flaring and venting the gas[191]. This is estimated to reduce the remaining emissions by half, leading to an MRP of 90% in 2100[7,8].

Emission reduction measures for natural gas mainly involve reducing emissions during transmission and distribution and controlling emissions during extraction, notably from unconventional gas wells. This is realized by: reducing leakage rates to levels currently observed in Western Europe, North America and Japan, and replacing grey cast iron pipes with PE and PVC networks[8]. Based on a case study of Russia, (2010)[192] estimated that up to 60% of distribution emissions can be reduced in 2030.

Consistent with this projection, in the GAINS dataset, the overall MRP for natural gas production and distribution is estimated at 62% in 2050. An important limitation for bringing emissions further down is the late identification of leaks. A promising, yet not employed and fully tested technology is optical gas imaging (OGI) in periodic leak detection and repair (LDAR) programs. Here, infrared cameras are used to scan large gas infrastructures for leakages and promptly address the major ones to be cost-effective. The technology can be broadly used and is estimated to help reduce net emissions by 60%-80%[193].

Industry and waste

CH_4 Domestic sewage and wastewater

Wastewater CH_4 emissions, generated by anaerobic bacteria, can be reduced by 1) Anaerobic digestion combined with CH_4 collection 2) Aerobic wastewater treatment 3) Replacing latrines and disposal by wastewater treatment plants (WWTP). For all reduction measures, biological CH_4 removal is commonly applied. A meta-study[194] found that aerobic biofilters, which are most commonly used, have the highest reduction efficiencies (REs); in 9 studies, REs of 50% to 100% was found (average = 75.4%). This is in line with [195] who found a reduction potential of 76.5%. The main problem for the removal of wastewater CH_4 is the low implementation potential of WWTPs that can reach maximum reductions. To a limited extent, a CH_4 price can function as a co-beneficial incentive to build WWTP infrastructure, next to health and sanitation. However, [196] found that even without a sewage system, composting toilets and biogas digesters can in principle have reduction efficiencies of up to 100%. The marginal costs of a composting toilet (calculated as the additional cost of a CH_4 mitigation technology beyond the cost of a pit latrine) are estimated at 169-3421 $(2010)/tCeq (large range depending on location). The global MRP in the US-EPA MACs is found to be 36% in 2030. Similar to the assumptions for landfills CH_4, technological progress is simulated by an increase in reduction potential of 2% every 5 years.

N_2O Transport

In transportation, the main N_2O sources are cars with a catalyst converter[197]. In these vehicles, the catalytic converters convert toxic gases and pollutants in exhaust gas such as CO, unburned hydrocarbons, and NO_x to less harmful pollutants or more inert gases such as CO_2, H_2O, N_2O, and N_2 through catalytic redox reactions[198,199]. Apart from a switch in the fuel or propulsion system, the key reduction measure is the implementation of low-N2O catalytic converters[11]. We applied the same assumptions as [11]. Implications of this assumption are expected to be relatively small, as the emission source is small. Short-term costs and reduction potentials are based on [13].

N_2O Nitric acid and Adipic acid

N_2O emissions from industry are mainly generated as a by-product of the production processes of adipic and nitric acid. For both processes, reduction potentials are found to be very high, at very low costs, when applying thermal or catalytic reduction. This study's MAC curves provide short-term reduction potentials and costs based on [3], which estimated the reduction potentials of adipic and nitric acid at 86%. N_2O from adipic acid production is assumed to be fully abatable in 2050 and 2100. While several studies estimate reduction potentials of 98%-99% at costs of 1-11$(2010)/tCeq[11,133,200,201], recent studies also found that emissions can be reduced by 100%[202,203], at high temperatures (600dC). It is assumed that this can be achieved at 50$(2010)/tCeq. The default MRP of N_2O from adipic acid

production is assumed to be 94% in 2050 and 2100 in the middle of the road estimate, based on the best available technology from [133]. This reduction potential is higher than found by [204] (80%) and [205] (80-90%), but deemed likely due to the very low cost of the technology (2$(2010)/tCeq). It is assumed that the MRP (including full technology implementation) can be achieved at 50$(2010)/tCeq.

CH_4 Landfills

Anaerobic bacteria generate CH_4 while degrading organic wastes deposited in landfills. Reduction options include 1) CH_4 collection and flaring 2) CH_4 capture for energy use 3) Biological processing 4) Waste diversion through recycling or incineration of organic waste (US-EPA, 2013). The global MRP of all landfill measures combined in the US-EPA (2019) dataset is found to be 51% in 2050, which excludes technological progress beyond the current state and full implementation of measures in all regions. Furthermore, the role of waste diversion and the banning of landfill of untreated organic waste is gaining in importance, especially in the EU where it is currently in use in several EU countries and about to be introduced in others (EC, 2015), with the potential to almost fully reduce CH_4 emissions. In addition, biological processing has seen several developments that could potentially increase future reductions. Methanotrophic bacteria placed in layers can in ideal conditions (e.g., low CH4 concentrations) remove generated CH_4 by 95%-100% by using methane as carbon and energy sources [206-208]. Covering low CH4 producing landfills with vegetated soils has led to similar reductions at very low costs (29 to 58 $(2010)/tCeq)[209]. Covering low CH_4 with compost has led to similar reductions as well because it enables the optimization of oxidizing methane into carbon dioxide[210]. After 2050, complementary to the US-EPA MACs, we assume a default increase in reduction potential of 2% per 5 years to simulate the effect of additional waste diversion and biological treatment, which we adopted in this study. Although such options might not be suitable for all landfill conditions, it is considered likely that at medium high costs, emission reductions converge to the maximum in all regions. The global MRP of 2050 obtained from GAINS data is 51%.

Fluorinated gases

The default MAC curves in this study are directly based on ref. [18]. The description in the following three paragraphs (on HFCs, PFCs and SF_6) are therefore copied from that paper's supplementary information. The focus in this study is on understanding any possible considerations to lower the (high) default reduction potentials, and to analyze the consequences of a lower potential in the scenario analysis.

Hydrofluorocarbons (HFCs)

HFCs are mainly used as propellant in foams and refrigerant in a wide range of cooling applications and can be emitted during and after use. In addition, HFC23 is emitted as a by-product in the production of HCFC-22, used as feedstock in industry and as refrigerant, although the latter use is being phased-out in accordance with the Montreal Protocol. In an extensive study, Schwarz, et al. [24] identified very high reduction potentials for all HFC species and applications, at low costs and equal functional performance of alternative solutions; HFCs used for foam blowing can be 100% substituted by zero GWP substances for estimated costs of 52 $/tC. The by-product HFC23 can very effectively be thermally destructed (estimated at 98% in 2100) at very low costs (below 2 $(2010)/tCeq)[211]. HFCs for other applications (mainly refrigeration related, but also use as solvents, aerosols and firefighting agents) can mainly be reduced by substitution with (near) zero GWP substances, complemented by better sealed systems and recovery after use, which is estimated to lead to a reduction efficiency of 97% at 258 $(2010)/tCeq.
The likelihood of realizing the emission reductions has increased due to the 2016 Kigali Amendment to the Montreal protocol, in which all countries in the United Nations agreed to ambitious HFC abatement towards 2043.

Perfluorocarbons (PFCs)

PFCs are largely emitted as a by-product of primary aluminum production, followed by the use and loss of PFCs in semiconductor manufacturing. In smaller quantities, PFCs are emitted when used as solvents, refrigerants and firefighting agents. For PFC mitigation in aluminum production, several options exist. Point-Feed Prebake technology (PFPB) leads to high reductions at moderate costs. Following Lucas et al. (2007), these are assumed to lead to a MRP of 80% in 2050 at 635 $(2010)/tCeq. A reduction efficiency of 100% is in principle possible, when applying Inert Anode (IA) technology, but this is yet to be fully developed and likely to come at a much higher cost[27]. For 2100, an overall MRP of 90% is assumed, which includes technological improvement of presently available PFPB and partial introduction of new technologies such as IA. PFC emissions from semiconductor manufacturing can be near 99% mitigated, either by thermal destruction or substitution[11,27]. An overall MRP is assumed of 80% and 100% in 2050 and 2100, respectively (assuming full implementation in the latter year), at 254 $(2010)/tCeq. For all other PFC sources, the main mitigation option is substitution by zero GWP substances. The MRP in 2100 is assumed to be slightly lower (95%), due to a larger diversity in sources, also at 254$(2010)/tCeq.

Sulphur hexafluoride (SF6)

SF_6 emissions occur during the production and use of electrical switchgear equipment and the decommissioning of sound-proof windows, and is released as a by-product of industrial activities, mainly of magnesium production. For SF_6, the same assumptions have been applied as by Lucas, et al. [11], as they closely resemble estimates found in recent work[27]. Emissions from the production, as well as the decommissioning of, electrical equipment can be mitigated by "good practice": improved recovery and recycling, reduced leakage and improved handling. The MRP is estimated at 80% and 90% in 2050 and 2100, respectively at 317 $(2010)/tCeq. Emissions from magnesium production can be reduced by replacing SF_6 as a protective gas during magnesium casting and is estimated to lead to an MRP of 90% in 2050 and 2100 at 127 $(2010)/tCeq.

Mitigation options for other sources also involve good practice or even the ban of SF_6 use in the case of sound-proof windows[27]. The MRP is estimated at 90% and 100% in 2050 and 2100, respectively, at 508 $(2010)/tCeq.

S7 Input parameters Monte Carlo analysis

Table S7.1: Main input values for the Monte Carlo analysis. See further description of the table in this section.

Emission source	Reduction measure	Technical Applicability (mean)	Reduction efficiency (mean)	Correction for overlap	Costs (2020$ /tCO_2eq)	Implementation potential (2050)	Implementation potential (2100)	Technological progress (2050)	Technological progress (2100)
Rice CH_4	Direct seeding	75%	20%	100%	0	70%	100%	90%	80%
	Replace urea with ammonium sulphate	75%	24%	97%	20	70%	100%	90%	80%
	Straw compost	50%	48%	94%	188	70%	100%	90%	80%
	Alternate flooding / drainage	40%	57%	89%	196	70%	100%	90%	80%
	Addition of phospogypsum	75%	39%	85%	510	70%	100%	90%	80%
Enteric Fermentation CH_4	Addition of nitrate to the feed	60%	23%	20%	142	90%	100%	90%	100%
	Genetic selection and breeding	75%	19%	20%	0	90%	100%	90%	100%
	Adding tannins as a food supplement	75%	20%	50%	20	90%	100%	90%	100%
	Grain processing	75%	23%	20%	66	90%	100%	90%	100%
	Improved health monitoring and illness prevention	75%	11%	100%	0	90%	100%	90%	100%
	Seaweed	75%	62%	50%	284	90%	100%	90%	100%
Fertilizer N_2O	Nitrification inhibitors	100%	40%	50%	42	70%	100%	90%	100%
	Improved land manure application	45%	19%	70%	0	70%	100%	90%	100%
	Irrigation practices	63%	44%	30%	530	70%	100%	90%	100%
	Biochar	80%	29%	40%	292	70%	100%	90%	100%
	Spreader maintenance	100%	28%	70%	65	70%	100%	90%	100%
	Improved agronomy practices	45%	25%	50%	6	70%	100%	90%	100%
	No tillage	63%	35%	100%	0	70%	100%	90%	100%
Manure CH_4	Storage duration	90%	57%	100%	40	50%	100%	90%	100%
	Anaerobic digestion - warm	90%	63%	20%	23	50%	100%	90%	100%
	Anaerobic digestion - cold	90%	45%	20%	23	50%	100%	90%	100%
	Reduced dietary protein	50%	40%	20%	93	50%	100%	90%	100%
	Storage covering	50%	82%	20%	110	50%	100%	90%	100%
	Manure acidification	50%	43%	30%	197	50%	100%	90%	100%
	Housing systems and beddings	50%	65%	20%	201	50%	100%	90%	100%
	Maximum reduction potential				0				
Manure N_2O	Reduced dietary protein	100%	35%	20%	40	50%	70%	90%	100%
	Decreased manure storage time	90%	52%	100%	34	50%	70%	90%	100%
	Manure storage covering	50%	26%	30%	114	50%	70%	90%	100%
	Improved animal housing systems and bedding	100%	53%	20%	93	50%	70%	90%	100%
	Anaerobic digestion	50%	45%	20%	110	50%	70%	90%	100%
	Acidification	100%	44%	20%	197	50%	70%	90%	100%
Delta values		±30% points	min-max	±30% points	±80%	±30 %points	±30 %points	10% points	10% points

Table S7.1 provides the main input values for the Monte Carlo analysis. The **TA** values shown are the maximum regional TA values, other regional values may be lower. TA values for CH_4 manure anaerobic digester (90%) applies to the TA of the combination of the two types (warm, cold, which are mutually exclusive). Regional differences in TA are assumed for Enteric Fermentation and CH_4 manure, based on the GAINS model global CH_4 mitigation potentials for livestock in 2030 and 2050[22]. Note, that this comparison is only indicative, since the GAINS model provides short-term technical reduction estimates and excludes more speculative factors related to future technological change and removal of barriers). We differentiated regions based on regional maximum feasible reductions under equal reduction efficiency assumptions across regions (hence, differences in reductions largely indicate differences in technical applicability, mostly related to differences in climate and farming systems). We assume default TA values for regions with relative reductions of >20%, a 30%point reduction in TA for relative reductions between 5% and 20% and a 50%point reduction for relative reductions under 5%. Regions that were assigned a low TA value for CH_4 enteric fermentation are: North Africa, East Africa, South Africa, Turkey, the Middle East, India, Korea, Southeast Asia, Indonesia, the rest of south Asia, rest of south Africa. Regions that got medium TA values for CH_4 enteric fermentation are: Russia and China. Regions that were assigned the default TA values for CH_4 enteric fermentation are: Canada, USA, Mexico, Central America, Brazil, rest of South America, West Europe, Central Europe, Japan and Oceania. Regions that got low TA values for CH_4 manure are: North Africa, West Africa, East Africa, South Africa, Turkey, Ukraine, Kazakhstan, Middle East, India, Korea, South East Asia, Indonesia, rest of South Asia, rest of south Africa. Regions that got medium values for CH_4 manure are: Mexico, Central America, Brazil, rest of south America, Russia. The TA value for N_2O fertilizer No tillage is based on [212] (worldwide adoption). Irrigation practices is estimated based on an OECD study into water use and irrigation in agriculture[213]. Since irrigation was already integrated in several countries, the technical applicability was estimated at 63%. N_2O manure anaerobic digestion and acidification are assumed to have the same TA values as CH_4 manure.

Regional differences in **RE** are implemented in the anaerobic digester for CH_4 manure. Regional differences in reduction efficiency are due to the different impact of anaerobic digester on CH_4 emissions for warm and cold regions. Regions that are assumed cold are: Canada, USA, West Europe, Central Europe, Turkey, Ukraine, Russia. Regions that are assumed warm: Mexico, Central America, Brazil, Rest of South America, North Africa, West Africa, East Africa, South Africa, Kazakhstan, Middle East, India, Korea, China, Southeast Asia, Indonesia, Japan, Oceania, Rest of South Asia, Rest of South Africa.

Correction for overlap. For the measures N_2O anaerobic digestion and N_2O manure acidification the values were obtained from the study of Aberdeen[23] (see also [18] for a quantitative description). Regarding the newly added measures in this study: For biochar no overlap was assumed with irrigation practices, improved agronomy, spreader maintenance and with no tillage. Low overlap was assumed with improved land manure and medium overlap with nitrification inhibitors. For no tillage, no overlap was assumed with nitrification inhibitors, spreader maintenance, and biochar. Low overlap was assumed with improved land manure. Medium overlap was assumed with irrigation practices and improved agronomy. For irrigation practices, it was assumed that no overlap occurs with nitrification inhibitors, spreader maintenance and biochar. Low overlap was assumed with improved land manure application and medium overlap was assumed with no tillage. For seaweed, no overlap was assumed with grain processing, genetic selection and improved health monitoring. Low overlap was assumed with adding nitrate and adding tannins because these are both also feed supplements. For solid-liquid separation, low overlap was assumed with manure storage duration, housing systems and beddings, manure storage covering, manure acidification and reduced dietary protein. High overlap was assumed with anaerobic digestion.

Costs: only “Rest of world” values shown. See below for a regional specifications, underlying assumptions and references.

Table S7.2: **Measure-specific cost estimates, assumptions and references**. Unless otherwise specified, costs are given for the “rest of world” (ROW) regions and in 2020$/t$CO_2$ eq. See below for regional specification. Costs are implemented in the model on a 2005$ basis (assumed 2005-2020 conversion: 1.325)

Emission source	Costs	Reference	Assumptions / comments
**CH_4 enteric fermentation**			
Nitrate	142	[50,214]	
Tannins	20	[215]	
Grain processing	66		Estimate (roughly mean of supplements)
Genetic selection	0	[59]	Now zero costs, since it is currently aimed at profit maximization through increased production
Improved health monitoring	0	[50,215]	
Seaweed (Asparagopsis taxiformis)	284		No information is available. Assumed twice as expensive as adding nitrate.
**CH_4 rice**			
Alternate flooding and drainage	196	[64,82]	
Direct seeding	83	[59]	Cost estimate for Asian regions (0 in ROW)
Addition of phosphogypsum	81	[59]	Cost estimate for Asian regions (510 in ROW)
Replace urea with ammonium sulphate	2	[59]	Cost estimate for Asian regions (19 in ROW)
Straw mitigation	37	[59,60]	
**CH_4 manure**			
Manure acidification	110	[50]	
Anaerobic digestion (cool)	0	[59]	
Anaerobic digestion (hot)	45	[59]	
Solid-liquid separation	201	[216,217]	
Manure storage: duration	40		Estimate
Housing and bedding	791	[218]	This is for Germany. Also, in [10,114,219], some additional mitigation is assumed above 100$/t$CO_2$.
Manure storage covering	186	[218]	Average lowest cost straw covering Dairy/pigs
**N_2O fertilizer**			
Sub-optimal fertilizer applications	182	[59]	OECD estimate.
Nitrification inhibitors	235	[50,122,220]	OECD estimate. [50] show the UK MAC curve update from [122]
Improved land manure application	0	[122]	
Irrigation practices	530	[221-223]	The carbon footprint of rice is approximately 2504 kg CO_2 eq/ha[222]. Drip irrigation costs approximately $5000 / ha / 5 yrs[221]. This gives a value for the costs of 400 $ / t CO2 eq. This is equal to the value found in [223].
Biochar	292		The costs of biochar were, very roughly, estimated at 220 $ / tCO_2 eq.
Spreader maintenance	0	[59]	OECD estimate.
Improved agronomy practices	5	[50,122,219]	OECD estimate. [50] show the UK MAC curve update from [122]
No tillage	0	[223]	
**N_2O manure**			
Reduced dietary protein	114	[215]	Supplements high
Decrease manure storage time	40		Estimated at 30$/t$CO_2$ eq.
Manure storage covering	186	[218]	Average lowest cost straw covering Dairy/pigs
Housing and bedding	197	[218]	This is for Germany. Also, in ref. [10,114,219], some additional mitigation is assumed above 100$/t$CO_2$.
Manure acidification	110	[50]	UK case study (CH_4 manure focused)
Anaerobic digestion	34	[59]	Eastern Europe case study (CH_4 manure focused)

Table S7.3: Regional, measure-specific cost estimates. Unless otherwise specified, Costs in 2020$/t$CO_2$ eq. Costs are implemented in the model on a 2005$ basis (assumed 2005-2020 conversion: 1.325)

Emission source	Mitigation option	Canada	USA	Mexico	Central America	Brazil	Rest of South-America	North Africa	West Africa	East Africa
**CH_4 Rice**	Direct seeding	0	0	0	0	0	0	0	0	0
	Replace urea with ammonium sulphate	20	20	20	20	20	20	20	20	20
	Straw compost	188	188	188	188	188	188	188	188	188
	Alternate flooding / drainage	196	196	196	196	196	196	196	196	196
	Addition of phospogypsum	510	510	510	510	510	510	510	510	510
**CH_4 Enteric Fermentation**	Addition of nitrate to the feed	142	142	142	142	142	142	142	142	142
	Genetic selection and breeding	0	0	0	0	0	0	0	0	0
	Adding tannins as a food supplement	20	20	20	20	20	20	20	20	20
	Grain processing	66	66	66	66	66	66	66	66	66
	Improved health monitoring and illness prevention	0	0	0	0	0	0	0	0	0
	Seaweed	284	284	284	284	284	284	284	284	284
**N_2O Fertilizer**	Nitrification inhibitors	235	235	42	42	42	42	42	42	42
	Improved land manure application	0	0	0	0	0	0	0	0	0
	Irrigation practices	530	530	530	530	530	530	530	530	530
	Biochar	292	292	292	292	292	292	292	292	292
	Spreader maintenance	65	65	65	65	65	65	65	65	65
	Improved agronomy practices	6	6	6	6	6	6	6	6	6
	No tillage	0	0	0	0	0	0	0	0	0
**CH_4 Manure**	Storage duration	40	40	40	40	40	40	40	40	40
	Anaerobic digestion - warm	69	52	23	23	23	23	23	23	23
	Anaerobic digestion - cold	69	52	23	23	23	23	23	23	23
	Reduced dietary protein	93	93	93	93	93	93	93	93	93
	Storage covering	110	110	110	110	110	110	110	110	110
	Manure acidification	197	197	197	197	197	197	197	197	197
	Housing systems and beddings	201	201	201	201	201	201	201	201	201
**N_2O Manure**	Reduced dietary protein	40	40	40	40	40	40	40	40	40
	Decreased manure storage time	34	34	34	34	34	34	34	34	34
	Manure storage covering	114	114	114	114	114	114	114	114	114
	Improved animal housing systems and bedding	93	93	93	93	93	93	93	93	93
	Anaerobic digestion	110	110	110	110	110	110	110	110	110
	Acidification	197	197	197	197	197	197	197	197	197

Emission source	Mitigation option	South Africa	West Europe	Central Europe	Turkey	Ukraine	Kazachs tan	Russia	Middle East	India
CH_4 Rice	Direct seeding	0	0	0	0	0	0	0	0	0
	Replace urea with ammonium sulphate	20	20	20	20	20	20	20	20	20
	Straw compost	188	188	188	188	188	188	188	188	188
	Alternate flooding / drainage	196	196	196	196	196	196	196	196	196
	Addition of phospogypsum	510	510	510	510	510	510	510	510	510
CH_4 Enteric Fermentation	Addition of nitrate to the feed	142	142	142	142	142	142	142	142	142
	Genetic selection and breeding	0	0	0	0	0	0	0	0	0
	Adding tannins as a food supplement	20	20	20	20	20	20	20	20	20
	Grain processing	66	66	66	66	66	66	66	66	66
	Improved health monitoring and illness prevention	0	0	0	0	0	0	0	0	0
	Seaweed	284	284	284	284	284	284	284	284	284
N_2O Fertilizer	Nitrification inhibitors	42	235	235	235	235	235	235	235	42
	Improved land manure application	0	0	0	0	0	0	0	0	0
	Irrigation practices	530	530	530	530	530	530	530	530	530
	Biochar	292	292	292	292	292	292	292	292	292
	Spreader maintenance	65	65	65	65	65	65	65	65	65
	Improved agronomy practices	6	6	6	6	6	6	6	6	6
	No tillage	0	0	0	0	0	0	0	0	0
CH_4 Manure	Storage duration	40	40	40	40	40	40	40	40	40
	Anaerobic digestion - warm	23	23	23	34	60	60	60	23	34
	Anaerobic digestion - cold	23	23	23	34	60	60	60	23	34
	Reduced dietary protein	93	93	93	93	93	93	93	93	93
	Storage covering	110	110	110	110	110	110	110	110	110
	Manure acidification	197	197	197	197	197	197	197	197	197
	Housing systems and beddings	201	201	201	201	201	201	201	201	201
N_2O Manure	Reduced dietary protein	40	40	40	40	40	40	40	40	40
	Decreased manure storage time	34	34	34	34	34	34	34	34	34
	Manure storage covering	114	114	114	114	114	114	114	114	114
	Improved animal housing systems and bedding	93	93	93	93	93	93	93	93	93
	Anaerobic digestion	110	110	110	110	110	110	110	110	110
	Acidification	197	197	197	197	197	197	197	197	197

Emission source	Mitigation option	Korea	China	South East Asia	Indonesia	Japan	Oceania	Rest of South Asia	Rest of South Africa	Rest of the world
**CH_4 Rice**	Direct seeding	84	84	84	84	0	0	0	0	0
	Replace urea with ammonium sulphate	1	1	1	1	20	20	20	20	20
	Straw compost	37	37	37	37	188	188	188	188	188
	Alternate flooding / drainage	196	196	196	196	196	196	196	196	196
	Addition of phospogypsum	81	81	81	81	510	510	510	510	510
**CH_4 Enteric Fermentation**	Addition of nitrate to the feed	142	142	142	142	142	142	142	142	142
	Genetic selection and breeding	0	0	0	0	0	0	0	0	0
	Adding tannins as a food supplement	20	20	20	20	20	20	20	20	20
	Grain processing	66	66	66	66	66	66	66	66	66
	Improved health monitoring and illness prevention	0	0	0	0	0	0	0	0	0
	Seaweed	284	284	284	284	284	284	284	284	284
**N_2O Fertilizer**	Nitrification inhibitors	42	42	42	42	235	235	42	42	235
	Improved land manure application	0	0	0	0	0	0	0	0	0
	Irrigation practices	530	530	530	530	530	530	530	530	530
	Biochar	292	292	292	292	292	292	292	292	292
	Spreader maintenance	65	65	65	65	65	65	65	65	65
	Improved agronomy practices	6	6	6	6	6	6	6	6	6
	No tillage	0	0	0	0	0	0	0	0	0
**CH_4 Manure**	Storage duration	40	40	40	40	40	40	40	40	40
	Anaerobic digestion - warm	23	23	23	34	23	23	23	23	23
	Anaerobic digestion - cold	23	23	23	34	23	23	23	23	23
	Reduced dietary protein	93	93	93	93	93	93	93	93	93
	Storage covering	110	110	110	110	110	110	110	110	110
	Manure acidification	197	197	197	197	197	197	197	197	197
	Housing systems and beddings	201	201	201	201	201	201	201	201	201
**N_2O Manure**	Reduced dietary protein	40	40	40	40	40	40	40	40	40
	Decreased manure storage time	34	34	34	34	34	34	34	34	34
	Manure storage covering	114	114	114	114	114	114	114	114	114
	Improved animal housing systems and bedding	93	93	93	93	93	93	93	93	93
	Anaerobic digestion	110	110	110	110	110	110	110	110	110
	Acidification	197	197	197	197	197	197	197	197	197

S8 Marginal Abatement Cost curves, non-agricultural sources

Figure 8.1: Marginal abatement cost curves – Fossil energy sources – 2050 (left) and 2100 (right), global averages.

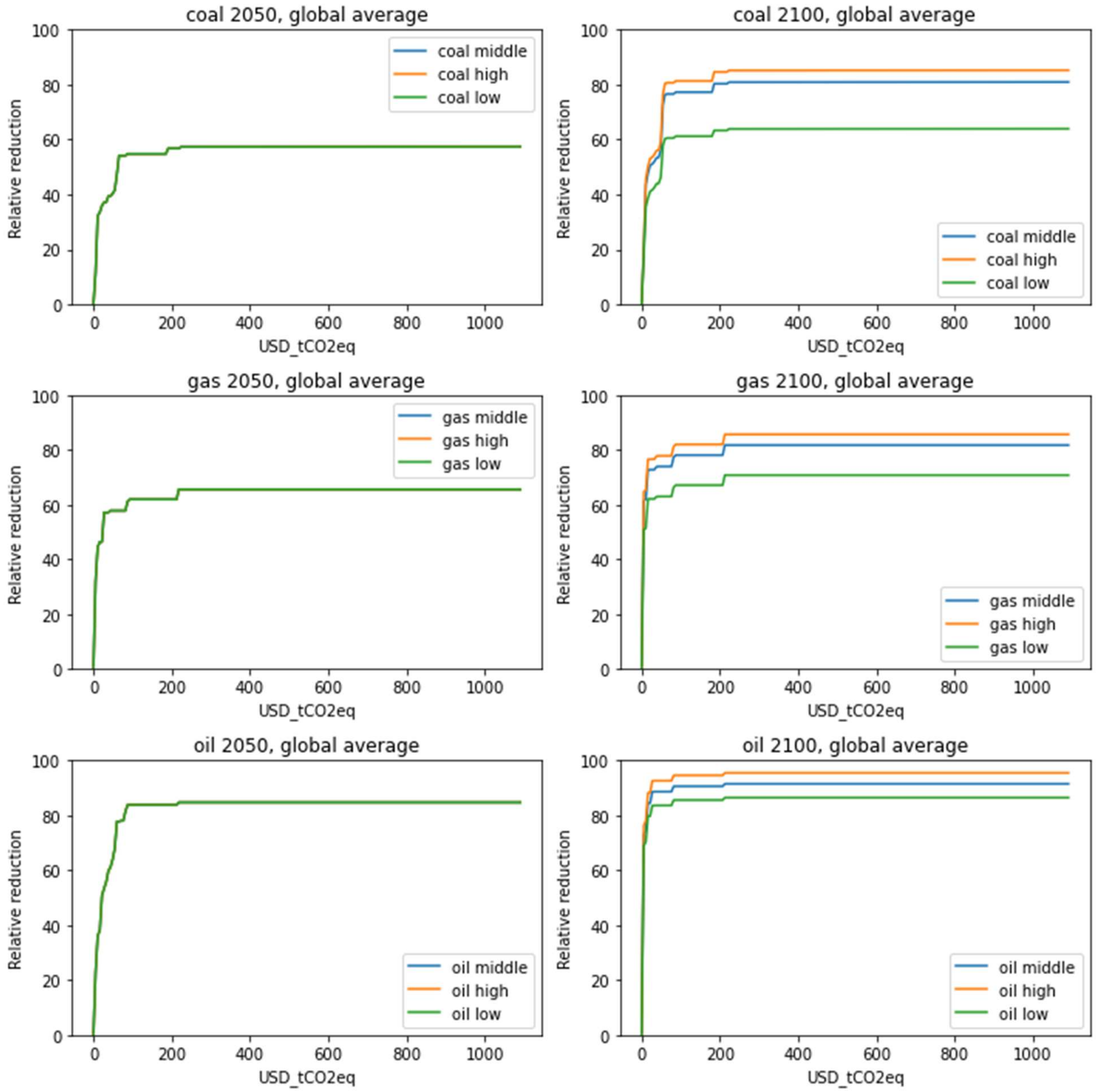

Figure 8.2: Marginal abatement cost curves – Waste and industry sources – 2050 (left) and 2100 (right), global averages.

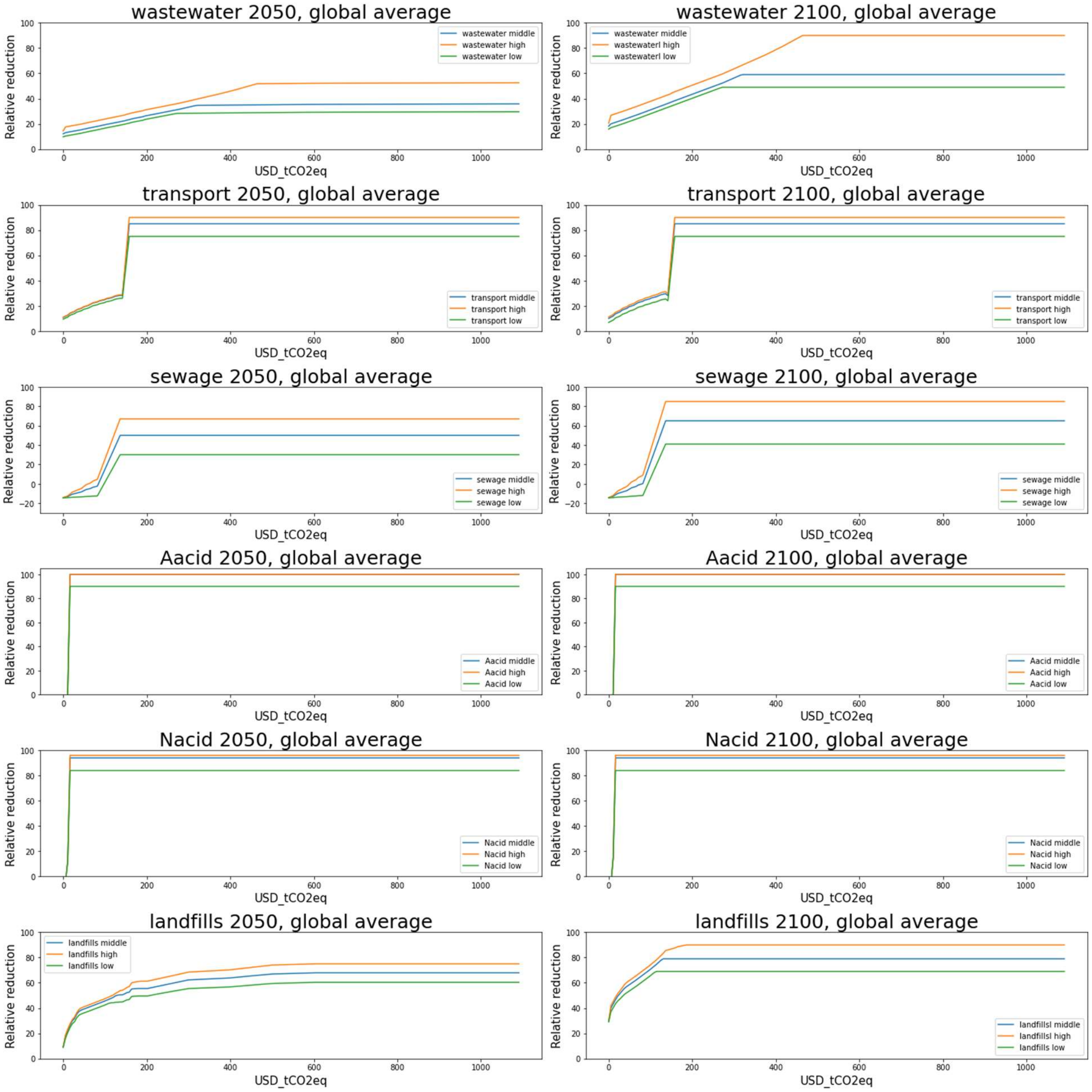

S9 Range in maximum reduction potentials

Table S9.1: Maximum reduction potentials (note, the MRPs for CH_4 coal/oil/gas, can be higher globally, since individual regions in the original dataset[25] can perform better than the set MRP. The MRP values for agricultural sources show the regional range.

Emission source	Default MRP 2050 (%)	MRP in 2050 (this study) (%)			Default MRP 2100 (%)	MRP in 2100 (this study) (%)		
	Harmsen et al. 2019	Pessimistic	Default	Optimistic	Harmsen et al. 2019	Pessimistic	Default	Optimistic
**CH_4**								
Coal	54	-	-	-	79	54	79	84
Oil	80	-	-	-	90	82	90	95
Gas	62	-	-	-	80	54	80	85
Landfills	75	51	61	66	90	69	79	90
Wastewater	62	30	50	67	90	49	59	90
Rice	61	41	59	78	77	57-58	70-71	82-83
Enteric fermentation with seaweed	-	-	-	41-58	-	-	-	47-65
Enteric fermentation without seaweed	41	8-15	18-26	-	50	18-24	28-36	-
manure	55	11-22	25-40	39-60	71	23-40	38-57	54-74
**N_2O**								
Transport	85	75	85	90	85	75	85	90
Aacid	100	90	100	100	100	90	100	100
Nacid	90	84	94	96	90	84	94	96
Fertilizer with biochar	-	-	-	69-70	-	-	-	74-75
Fertilizer without biochar	47	32	49	-	64	48	60-61	-
N2O manure	47	28	50	71	63	46	65	83
Sewage	50	30	50	57	65	41	65	85
**F-gases**								
HFCs - Refrigeration	96	90	96	96	96	92	96	96
HFCs - Foams	100	90	100	100	100	95	100	100
HFCs - Production of HCFC-22	90		90	90	98		98	98
HFCs - Other	97	90	97	97	97	95	97	97
PFCs - Aluminium production	80	90	80	80	90	93	90	90
PFCs - Semi-conductor production	80	54	80	80	99	80	99	99
PFCs - Other sources PFCs	80	77	80	80	95	77	95	95
SF6 - Production of electrical equipment	80	60	80	80	90	75	90	90
SF6- Use and decommissioning of elec. Eq.	80	65	80	80	90	75	90	90
SF6 - Magnesium production	90	75	90	90	90	75	90	90
SF6 - Other sources	90	70	90	90	100	80	100	100

S10 Scenario results: Emissions, Radiative forcing, Global mean temperature

This section presents the key scenario emission trajectories (including CO_2), as well as radiative forcing and global mean temperature (GMT) change profiles. Several relevant points should be mentioned here:

- Using the IPCC's scenario classification[224], the 1.5-degree scenarios are C2 scenarios (reaching 1.5-degrees after a temperature overshoot), with cumulative CO2 emission range of –90 to 620 Gt in the 2020-2100 period. These 1.5-degree scenarios fit within that cumulative CO_2 range with carbon budgets of 76 (1.5M) and 260 (1.5H). However, note that these scenarios are developed with the aim of having >66.6% chance of staying below 1.5 degrees, whereas the C2 category also allows 1.5-degree scenarios that have a 50% chance of staying below 1.5-degrees.
- The 2-degree scenarios are of category C3b (with a >66.6% chance of staying below 2 degrees, after following the NDCs until 2030). The carbon budgets in this study's scenarios also fall within the AR6 560–1050 range (see Figure 2 in the main text).
- CO_2 emission strategies are similar across the (SSP2-based) 1.5-degree or 2-degree scenarios. However, the trajectories differ in overall emission reductions, with higher non-CO_2 emission corresponding to lower CO_2 emissions and vice versa.
- Global F-gas emissions are expected to be reduced by >90% in all scenarios, including 2L, with pessimistic MAC assumptions. Hence, uncertainty in F-gas mitigation remains relatively small. The emission trajectories clearly show that CH_4 and N_2O emissions are much more uncertain, particularly from agriculture. This can therefore be considered the major factor influencing climate policy feasibility.
- This study does not find any significant impact of MAC uncertainty on peak warming. Maximum radiative forcing and GMT are very similar across the (SSP2-based) 2-degree scenarios or across the 1.5-scenarios. The main reason is a relatively low, short-term reduction potential, leading to relatively small differences between MACs in the short term (until 2030-2040, when maximum forcing is reached). Note however, that peak temperature is found to be slightly (0.02 degrees) lower in the 2H_SSP1 case, due to earlier allowed action (ratcheting up the NDCs) and lower (SSP1) baseline emissions.

Figure S10.1: Global CO_2 trajectories. Left: 2-degree scenarios, right: 1.5-degree scenarios

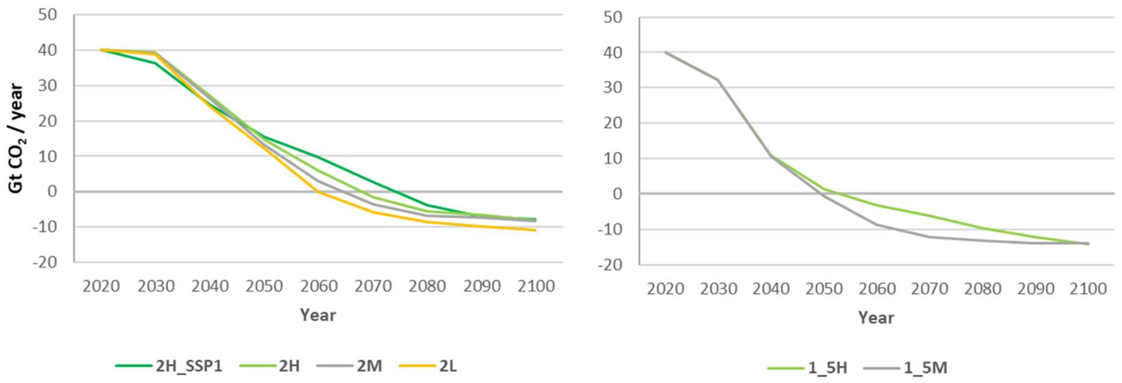

Table S10.1: Global CO_2 emissions (in Gt CO_2 / year)

	2020	2030	2040	2050	2060	2070	2080	2090	2100
**Base**	40.0	47.7	51.4	54.8	54.4	55.5	57.2	55.5	50.9
**2H_SSP1**	40.0	36.3	24.5	15.6	9.8	2.7	-3.9	-7.1	-7.9
**2H**	40.0	39.4	27.0	14.8	6.0	-1.5	-5.6	-6.6	-8.2
**2M**	40.0	39.1	26.3	13.3	3.0	-3.7	-6.9	-7.4	-8.3
**2L**	40.0	38.8	24.0	12.2	0.0	-5.9	-8.6	-9.9	-10.8
**1_5H**	40.0	32.3	10.8	1.3	-3.3	-6.2	-9.6	-12.2	-14.3
**1_5M**	40.0	32.2	10.7	-0.5	-8.8	-12.1	-13.2	-14.0	-14.0

Figure S10.2: Global CH_4 trajectories. Left: 2-degree scenarios, right: 1.5-degree scenarios. Baseline emissions are included as a comparison.

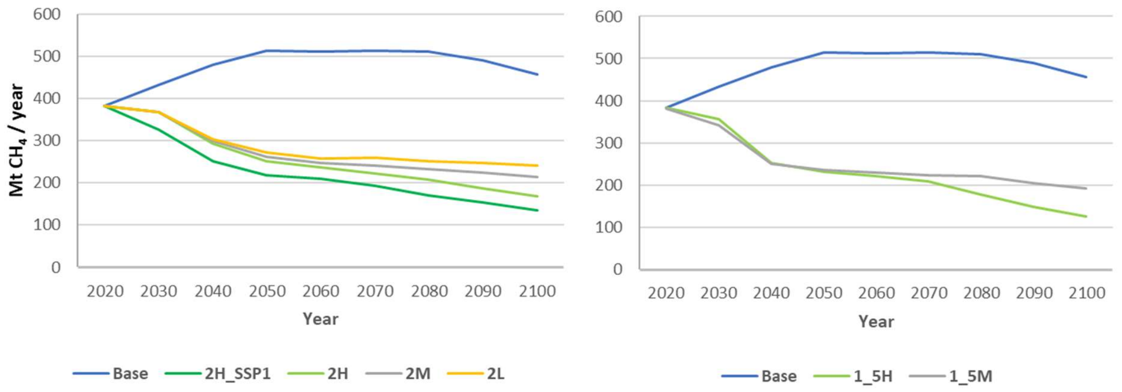

Table S10.2: Global CH_4 emissions (in Mt CH_4 / year)

	2020	2030	2040	2050	2060	2070	2080	2090	2100
**Base**	383	433	479	514	512	514	510	490	456
**2H_SSP1**	382	327	251	217	210	193	170	153	135
**2H**	383	367	293	252	236	222	207	187	169
**2M**	383	368	299	262	247	240	232	224	214
**2L**	383	368	304	271	258	259	252	248	240
**1_5H**	383	357	253	231	222	209	178	149	126
**1_5M**	383	341	252	236	229	224	221	205	192

Figure S10.3: Global N_2O trajectories. Left: 2-degree scenarios, right: 1.5-degree scenarios. Baseline emissions are included as a comparison.

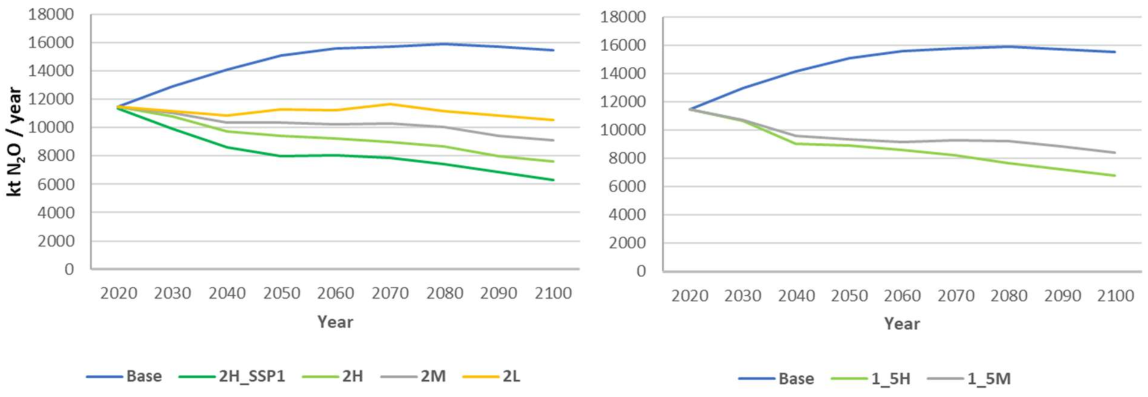

Table S10.3: Global N_2O emissions (in kt N_2O / year)

	2020	2030	2040	2050	2060	2070	2080	2090	2100
**Base**	11479	12943	14127	15070	15565	15739	15904	15717	15492
**2H_SSP1**	11358	9902	8598	7977	8036	7860	7440	6854	6291
**2H**	11479	10803	9736	9397	9218	8991	8669	8011	7605
**2M**	11479	11025	10335	10331	10218	10318	10035	9420	9081
**2L**	11479	11197	10847	11294	11209	11656	11181	10884	10535
**1_5H**	11479	10680	9014	8890	8571	8213	7643	7238	6796
**1_5M**	11479	10736	9565	9334	9175	9257	9204	8826	8377

Figure S10.4: Global F-gas trajectories. Left: 2-degree scenarios, right: 1.5-degree scenarios. Baseline emissions are included as a comparison.

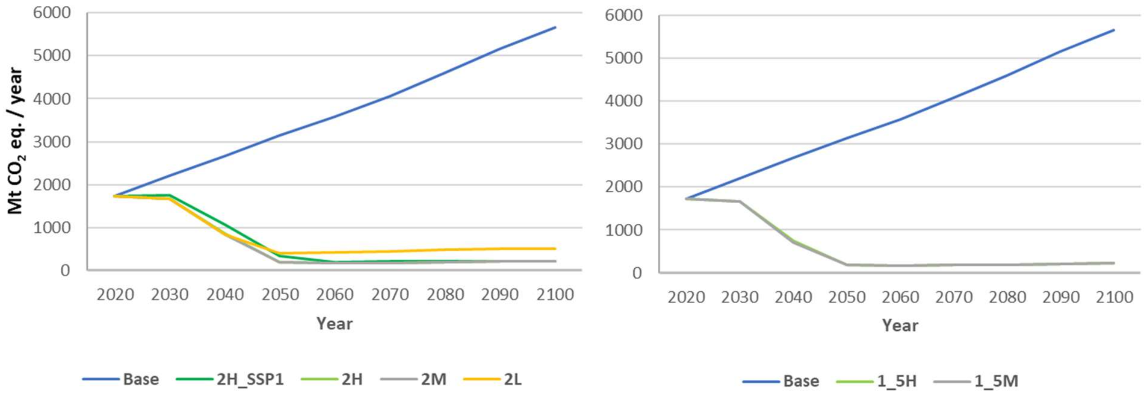

Table S10.4: Global F-gas emissions (in Mt CO_2 equivalents / year)

	2020	2030	2040	2050	2060	2070	2080	2090	2100
**Base**	1734	2206	2672	3138	3585	4067	4597	5165	5651
**2H_SSP1**	1734	1748	1062	335	204	225	225	212	218
**2H**	1734	1673	853	201	172	183	197	216	221
**2M**	1734	1666	831	199	172	183	197	216	221
**2L**	1734	1663	847	399	423	450	477	501	502
**1_5H**	1734	1673	742	186	172	183	197	214	220
**1_5M**	1734	1663	708	190	172	182	197	214	220

Figure S10.5: Radiative forcing profiles. Left: 2-degree scenarios, right: 1.5-degree scenarios

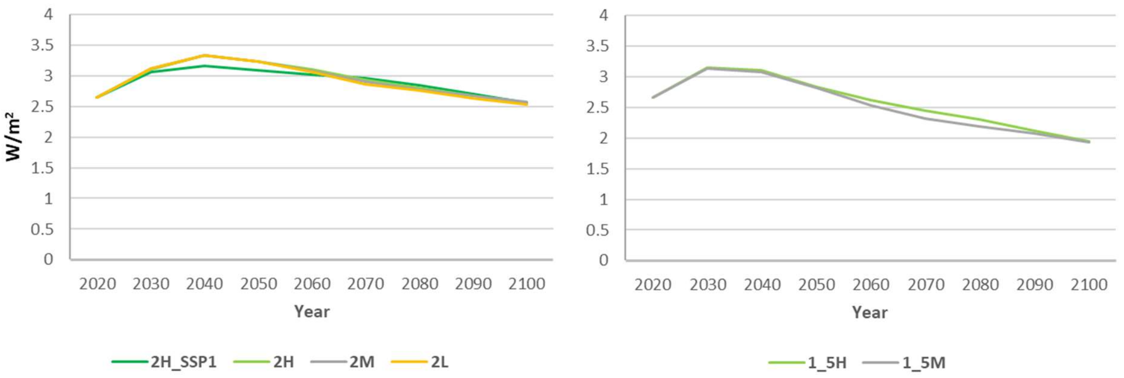

Table S11.0: Radiative forcing (in W/m^2)

	2020	2030	2040	2050	2060	2070	2080	2090	2100
**Base**	40.0	47.7	51.4	54.8	54.4	55.5	57.2	55.5	50.9
**2H_SSP1**	40.0	36.3	24.5	15.6	9.8	2.7	-3.9	-7.1	-7.9
**2H**	40.0	39.4	27.0	14.8	6.0	-1.5	-5.6	-6.6	-8.2
**2M**	40.0	39.1	26.3	13.3	3.0	-3.7	-6.9	-7.4	-8.3
**2L**	40.0	38.8	24.0	12.2	0.0	-5.9	-8.6	-9.9	-10.8
**1_5H**	40.0	32.3	10.8	1.3	-3.3	-6.2	-9.6	-12.2	-14.3
**1_5M**	40.0	32.2	10.7	-0.5	-8.8	-12.1	-13.2	-14.0	-14.0

Figure S10.6: Global mean temperature change profiles. Left: 2-degree scenarios, right: 1.5-degree scenarios

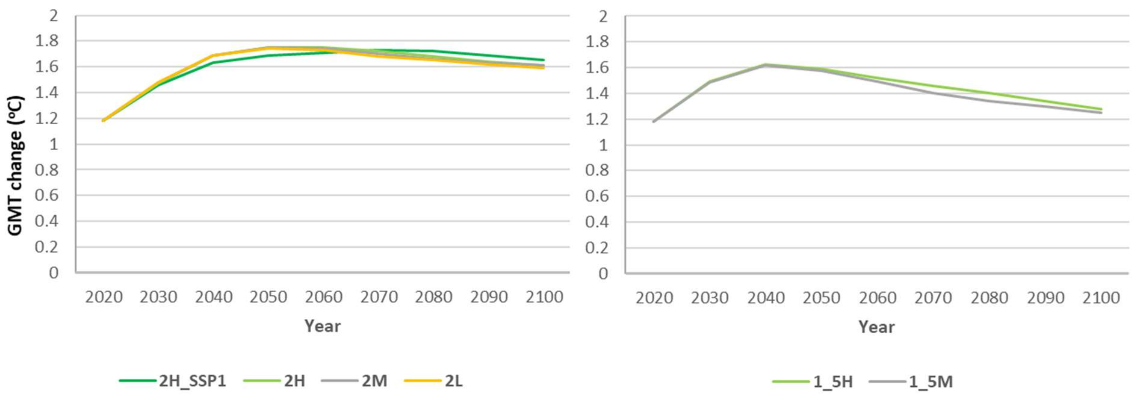

Table S10.6: Global mean temperature change (in ° Celsius)

	2020	2030	2040	2050	2060	2070	2080	2090	2100
**Base**	1.18	1.47	1.72	1.96	2.24	2.54	2.87	3.20	3.50
**2H_SSP1**	1.18	1.46	1.63	1.69	1.71	1.73	1.72	1.69	1.65
**2H**	1.18	1.47	1.69	1.75	1.75	1.72	1.68	1.64	1.61
**2M**	1.18	1.48	1.69	1.75	1.74	1.70	1.66	1.63	1.61
**2L**	1.18	1.48	1.69	1.74	1.73	1.68	1.65	1.62	1.59
**1_5H**	1.18	1.49	1.62	1.59	1.52	1.46	1.40	1.34	1.28
**1_5M**	1.18	1.49	1.62	1.58	1.49	1.40	1.34	1.30	1.25

S11 Emission source specific NCGG reductions in the scenarios

Table S11.1: Source-specific relative contribution to total NCGG reductions in 2100. Reductions compared to SSP2 in % reduced CO_2 equivalents based on AR4 GWP_{100}.

	2H_SSP1	2H	2M	2L	1.5H	1.5M	Average
CH_4	50%	49%	45%	46%	51%	47%	**48%**
N_2O	17%	16%	15%	13%	16%	15%	**16%**
HFCs	31%	33%	37%	38%	31%	35%	**34%**
PFCs	0.5%	0.5%	0.6%	0.5%	0.5%	0.6%	**0.5%**
SF_6	1.6%	1.7%	1.9%	1.7%	1.5%	1.8%	**1.7%**

Table S11.2: Emission source specific NCGG reductions in 2100. Infeasible scenarios are excluded here (1.5L and 2L_SSP3). Emission reductions in 2H_SSP1 also compared to the SSP2 baseline. F-gas emissions in CO_2 equivalents based on AR4 GWP_{100}.

	Emissions	Reductions					
	Base	2H_SSP1	2H	2M	2L	1.5H	1.5M
CH_4 AFOLU (Mt / %)	212	102 (48%)	77 (36%)	54 (25%)	45 (21%)	116 (55%)	77 (36%)
CH_4 Energy (Mt / %)	179	172 (96%)	162 (91%)	159 (89%)	149 (83%)	162 (90%)	158 (88%)
CH_4 waste (Mt / %)	65	47 (72%)	51 (78%)	30 (46%)	21 (32%)	51 (78%)	30 (46%)
N_2O AFOLU (kt / %)	12614	6700 (53%)	5877 (47%)	4597 (36%)	3564 (28%)	6495 (51%)	5034 (40%)
N_2O Energy (kt / %)	841	601 (71%)	359 (43%)	352 (42%)	325 (39%)	321 (38%)	306 (36%)
N_2O Waste (kt / %)	349	305 (87%)	293 (84%)	219 (63%)	130 (37%)	293 (84%)	219 (63%)
N_2O Industrial processes (kt / %)	1688	1595 (94%)	1515 (90%)	1513 (90%)	1451 (37%)	1546 (92%)	1547 (92%)
HFCs (Gt CO_2 eq. / %)	5157	4985 (97%)	4969 (96%)	4969 (96%)	4745 (92%)	4969 (96%)	4969 (96%)
PFCs (Gt CO_2 eq. / %)	89	80 (90%)	80 (90%)	80 (90%)	66 (75%)	80 (90%)	80 (90%)
SF_6 (Gt CO_2 eq. / %)	274	253 (92%)	249 (91%)	249 (91%)	207 (76%)	249 (91%)	249 (91%)

Supplementary References

1 Harmsen, M. *et al.* The role of methane in future climate strategies: Mitigation potentials and climate impacts. *Climatic Change* **163**, 1409–1425 (2020). https://doi.org:https://doi.org/10.1007/s10584-019-02437-2

2 Akashi, O. & Hanaoka, T. Technological feasibility and costs of achieving a 50 % reduction of global GHG emissions by 2050: mid- and long-term perspectives. *Sustainability Science* **7**, 139-156 (2012). https://doi.org:10.1007/s11625-012-0166-4

3 US-EPA. Global Non-CO2 Greenhouse Gas Emission Projections & Mitigation, 2015–2050. (United States Environmental Protection Agency Office of Atmospheric Programs (6207A), Washington, 2019).

4 Hyman, R. C., Reilly, J. M., Babiker, M. H., Masin, A. D. & Jacoby, H. D. Modeling non-CO2 greenhouse gas abatement. *Environmental Modeling and Assessment* **8**, 175–186 (2002).

5 US-EPA. Global Mitigation of Non-CO2 Greenhouse Gases. United States Environmental Protection Agency, EPA 430-R-06-005, Washington, D.C. . (2006).

6 Havlík, P. *et al.* Climate change mitigation through livestock system transitions. *PNAS* **111**, 3709–3714 (2014).

7 Höglund-Isaksson, L. Bottom-up simulations of methane and ethane emissions from global oil and gas systems 1980 to 2012. *Environmental Research Letters* **12** (2017).

8 Höglund-Isaksson, L. Global anthropogenic methane emissions 2005–2030: technical mitigation potentials and costs. *Atmos. Chem. Phys.* **12**, 9079-9096 (2012).

9 Havlik, P. *et al.* GLOBIOM documentation, International Institute for Applied Systems Analysis (IIASA). (2018).

10 US-EPA. United States Environmental Protection Agency (USEPA), Global Mitigation of Non-CO2 Greenhouse Gases: 2010-2030. (2013).

11 Lucas, P. L., Van Vuuren, D. P., Olivier, J. G. J. & Den Elzen, M. G. J. Long-term reduction potential of non-CO_2 greenhouse gases. *Environmental Science & Policy* **10**, 85 - 103 (2007).

12 Rao, S. & Riahi, K. The Role of Non-CO2 Greenhouse Gases in Climate Change Mitigation: Long-term Scenarios for the 21st Century. *The Energy Journal* **Special Issue #3**, 177-200 (2006).

13 GECS. Greenhouse Gas Emission Control Strategies - Research Project N° EVK2-CT-1999-00010, Thematic Programme : Environment and Sustainable Development of the DG Research Fifth Framework Programme. (2002).

14 Stehfest, E., van Vuuren, D. P., Kram, T., Bouwman , A. F. & (eds.). *Integrated Assessment of Global Environmental Change with IMAGE 3.0. Model description and policy applications*. *The Hague: PBL Netherlands Environmental Assessment Agency* (2014).

15 Van Vuuren, D. P. *et al.* The 2021 SSP scenarios of the IMAGE 3.2 model (Preprint Earth Arxiv, 2759). (2021). https://doi.org:https://doi.org/10.31223/X5CG92

16 Riahi, K. *et al.* The Shared Socioeconomic Pathways and their energy, land use, and greenhouse gas emissions implications: An overview. *Global Environmental Change* **42**, 153-168 (2017). https://doi.org:http://dx.doi.org/10.1016/j.gloenvcha.2016.05.009

17 Van Vuuren, D. P. *et al.* Energy, land-use and greenhouse gas emissions trajectories under a green growth paradigm. *Global Environmental Change* **42**, 237-250 (2017).

18 Harmsen, J. H. M. *et al.* Long-term marginal abatement cost curves of non-CO2 greenhouse gases. *Environmental Science & Policy* **99**, 136-149 (2019). https://doi.org:10.1016/j.envsci.2019.05.013

19 Hoesly, R. M. *et al.* Historical (1750–2014) anthropogenic emissions of reactive gases and aerosols from the Community Emissions Data System (CEDS). *Geoscientific Model Development* **11**, 369-408 (2018). https://doi.org:10.5194/gmd-11-369-2018

20 EC-JRC/PBL. European Commission, Joint Research Centre (EC-JRC)/Netherlands Environmental Assessment Agency (PBL). Emissions Database for Global Atmospheric

Research (EDGAR), release EDGAR v4.3.2 (1970 - 2012) of March 2016, http://edgar.jrc.ec.europa.eu. (2016).
21 Velders, G. J. M., Fahey, D. W., Daniel, J. S., Andersen, S. O. & McFarland, M. Future atmospheric abundances and climate forcings from scenarios of global and regional hydrofluorocarbon (HFC) emissions. *Atmospheric Environment* **123**, 200-209 (2015). https://doi.org:10.1016/j.atmosenv.2015.10.071
22 GAINSv4. *Greenhouse gas –Air pollution Interaction and Synergies Model* <http://gains.iiasa.ac.at/> (2019).
23 Smith, P. *et al.* Science-based GHG emissions targets for agriculture and forestry commodities. https://www.pbl.nl/sites/default/files/cms/publicaties/pbl-2016-science-based-greenhouse-gas-emissions-targets-for-agriculture-and-forestry-commodities-2856.pdf. (2016).
24 Schwarz, W., Gschrey, B., Leisewitz, A., Herold, A. & Gores, S. Preparatory study for a review of Regulation (EC) No 842/2006 on certain fluorinated greenhouse gases"; Final Report Prepared for the European Commission in the context of Service Contract No 070307/2009/548866/SER/C4; September 2011. (2011).
25 Höglund-Isaksson, L., Gómez-Sanabria, A., Klimont, Z., Rafaj, P. & Schöpp, W. Technical potentials and costs for reducing global anthropogenic methane emissions in the 2050 timeframe –results from the GAINS model. *Environmental Research Communications* **2** (2020). https://doi.org:10.1088/2515-7620/ab7457
26 Sun, Y. *et al.* Enhanced biological nitrogen removal and N2O emission characteristics of the intermittent aeration activated sludge process. *Reviews in Environmental Science and Bio/Technology* **16**, 761-780 (2017). https://doi.org:10.1007/s11157-017-9444-z
27 Purohit, P. & Höglund-Isaksson, L. Global emissions of fluorinated greenhouse gases 2005–2050 with abatement potentials and costs. *Atmos. Chem. Phys.* **17**, 2795-2816 (2017). https://doi.org:https://doi.org/10.5194/acp-17-2795-2017
28 Stanley, K. M. *et al.* Increase in global emissions of HFC-23 despite near-total expected reductions. *Nature Communications* **11** (2020). https://doi.org:10.1038/s41467-019-13899-4
29 Dickie, A. *et al.* Strategies for Mitigating Climate Change in Agriculture: Abridged Report. Climate Focus and California Environmental Associates, prepared with the support of the Climate and Land Use Alliance. Report and supplementary. (2014).
30 Hulshof, R. B. A. *et al.* Dietary nitrate supplementation reduces methane emission in beef cattle fed sugarcane-based diets. *J. Anim. Sci.* **90**, 2317–2323 (2012).
31 Van Zijderveld, S. M. *et al.* Persistency of methane mitigation by dietary nitrate supplementation in dairy cows. *J. Dairy Sci.* **94**, 4028–4038 (2011).
32 van Wyngaard, J. D. V., Meeske, R. & Erasmus, L. J. Effect of dietary nitrate on enteric methane emissions, production performance and rumen fermentation of dairy cows grazing kikuyu-dominant pasture during summer. *Animal Feed Science and Technology* **244**, 76-87 (2018). https://doi.org:10.1016/j.anifeedsci.2018.08.005
33 Petersen, H., Brask, Højberg, Poulsen, Zhu, Baral, and Lund. Dietary Nitrate for Methane Mitigation Leads to Nitrous Oxide Emissions from Dairy Cows. *Journal of Environmental Quality* **44**, 1063-1070 (2015). https://doi.org:10.2134/jeq2015.02.0107
34 Lee, C., Araujo, R. C., Koenig, K. M., Hile, M. L., Fabian-Wheeler, E. E., & Beauchemin, K. A. Effects of Feeding Encapsulated Nitrate to Beef Cattle on Ammonia and Greenhouse Gas Emissions from Their Manure in a Short-Term Manure Storage System. *Journal of Environmental Quality* **45**, 1979-1987 (2016).
35 Alemu, A. W., Romero-Perez, A., Araujo, R. C. & Beauchemin, K. A. Effect of Encapsulated Nitrate and Microencapsulated Blend of Essential Oils on Growth Performance and Methane Emissions from Beef Steers Fed Backgrounding Diets. *Animals (Basel)* **9** (2019). https://doi.org:10.3390/ani9010021

36 Villar, L., Hegarty, R., Van Tol, M., Godwin, I., & Nolan, J. Dietary nitrate metabolism and enteric methane mitigation in sheep consuming a protein-deficient diet. *Animal production science* **60**, 232-241 (2019).
37 Bell, M. J., Wall, E., Russell, G., Morgan, C. & Simm, G. Effect of breeding for milk yield, diet and management on enteric methane emissions from dairy cows. *Animal production science* **50**, 817 -826 (2010).
38 Jonker, A., Hickey, S., Pinares-Patiño, C., McEwan, J., Olinga, S., Díaz, A., ... & Rowe, S. Sheep from low-methane-yield selection lines created on alfalfa pellets also have lower methane yield under pastoral farming conditions. *Journal of animal science* **95**, 3905-3913 (2017).
39 Habib, G., & Khan, A. A. Assessment and mitigation of methane emissions from livestock sector in Pakistan. *Earth Systems and Environment* **2**, 601-608 (2018).
40 MacLeod, M., Leinonen, I., Wall, E., Houdijk, J., Eory, V., Burns, J., ... & Gómez-Barbero, M. Impact of animal breeding on GHG emissions and farm economics. *Publications Office of the European Union.* (2019).
41 de Haas, Y., Veerkamp, R. F., de Jong, G. & Aldridge, M. N. Selective breeding as a mitigation tool for methane emissions from dairy cattle. *Animal* **15 Suppl 1**, 100294 (2021). https://doi.org:10.1016/j.animal.2021.100294
42 Adejoro, F. A., Hassen, A., & Akanmu, A. M. Effect of Lipid-Encapsulated Acacia Tannin Extract on Feed Intake, Nutrient Digestibility and Methane Emission in Sheep. *Animals* **9**, 863 (2019).
43 Alves, T. P., Dall-Orsoletta, A. C., & Ribeiro-Filho, H. M. N. The effects of supplementing Acacia mearnsii tannin extract on dairy cow dry matter intake, milk production, and methane emission in a tropical pasture. . *Tropical animal health and production* **49**, 1663-1668 (2017).
44 Hristov, A. N. *et al. Mitigation of greenhouse gas emissions in livestock production – A review of technical options for non-CO2 emissions. Edited by Pierre J. Gerber, Benjamin Henderson and Harinder P.S. Makkar. FAO Animal Production and Health Paper No. 177. FAO, Rome, Italy*. (2013).
45 Nayak, D. *et al.* Management opportunities to mitigate greenhouse gas emissions from Chinese agriculture. *Agriculture, Ecosystems and Environment* **209**, 108-124 (2015).
46 Perna, F., Vásquez, D. C. Z., Gardinal, R., Meyer, P. M., Berndt, A., Friguetto, R. T. S., ... & Rodrigues, P. H. M. Short-term use of monensin and tannins as feed additives on digestibility and methanogenesis in cattle. *Revista Brasileira de Zootecnia* **49** (2020).
47 Corona, L., Owens, F. N., & Zinn, R. A. Impact of corn vitreousness and processing on site and extent of digestion by feedlot cattle. *Journal of animal science* **84**, 3020-3031 (2006).
48 Hales, K. E., N. A. Cole, and J. C. MacDonald. Effects of corn processing method and dietary inclusion of wet distillers grains with solubles on energy metabolism, carbon−nitrogen balance, and methane emissions of cattle,. *Journal of animal science* **90**, 3174-3185 (2012).
49 Hales, K. E., & Cole, N. A. . Hourly methane production in finishing steers fed at different levels of dry matter intake. *Journal of animal science* **95**, 2089-2096 (2017).
50 Eory, V. *et al.* ClimateXChange study: On-farm technologies for the reduction of greenhouse gas emissions in Scotland. (2016).
51 MacLeod, M., Eory, V., Wint, W., Shaw, A., Gerber, P. J., Cecchi, G., ... & Robinson, T. Assessing the Greenhouse Gas Mitigation Effect of Removing Bovine Trypanosomiasis in Eastern Africa. *Sustainability* **10**, 1633 (2018).
52 Statham, J. M., Scott, H., Statham, S., Acton-RAFT, J., & Williams, A.G. (2020). Dairy Cattle Health and Greenhouse Gas Emissions Pilot Study : Chile , Kenya and the UK. Dairy Cattle Health and Greenhouse Gas Emissions Pilot Study: Chile, Kenya and the UK. (2020).
53 Li, X. *et al.* Asparagopsis taxiformis decreases enteric methane production from sheep. *Animal Production Science* **58**, 681-688 (2016).
54 Machado, L., Magnusson, M., Paul, N. A., Kinley, R., de Nys, R., & Tomkins, N. Dose-response effects of Asparagopsis taxiformis and Oedogonium sp. on in vitro fermentation and methane production. *Journal of applied phycology* **28**, 1443-1452 (2016).

55 Roque, B. M., Salwen, J. K., Kinley, R., & Kebreab, E. . Inclusion of Asparagopsis armata in lactating dairy cows' diet reduces enteric methane emission by over 50 percent. *Journal of Cleaner Production* **234**, 132-138 (2019).
56 Stefenoni, H. A., Räisänen, S. E., Cueva, S. F., Wasson, D. E., Lage, C. F. A., Melgar, A., ... & Hristov, A. N. . Effects of the macroalga asparagopsis taxiformis and oregano leaves on methane emsission, rumen fermentation, and lactational performance of dairy cows. *Journal of dairy science* **104**, 4157-4173 (2021).
57 Charles G. Brooke, B. M. R., Claire Shaw, Negeen Najafi, Maria Gonzalez, Abigail Pfefferlen, Vannesa De Anda, David W. Ginsburg, Maddelyn C. Harden2, Sergey V. Nuzhdin, Joan King Salwen, Ermias Kebreab, Matthias Hess. Methane reduction potential of two pacific coast macroalgae during in vitro ruminant fermentation. *Frontiers in Marine Science* **7** (2020).
58 Kinley, R. D., Nys, R. d., Vucko, M. J., Machado, L. & Tomkins, N. W. The red macroalgae Asparagopsis taxiformis is a potent natural antimethanogenic that reduces methane production during in vitro fermentation with rumen fluid. *Animal Production Science* **56**, 282-289 (2016).
59 Graus, W. J., Harmelink, M. & Hendriks, C. Marginal GHG-Abatement Curves for Agriculture. *Ecofys report, EEP030339, April 2004* (2004).
60 Launio, C. C., Asis, C. A., Manalili, R. G. & Javier, E. F. Cost-effectiveness analysis of farmers' rice straw management practices considering CH4 and N2O emissions. *J Environ Manage* **183**, 245-252 (2016). https://doi.org:10.1016/j.jenvman.2016.08.015
61 Romasanta, R. R., Sander, B. O., Gaihre, Y. K., Alberto, M. C., Gummert, M., Quilty, J., ... & Wassmann, R. . How does burning of rice straw affect CH4 and N2O emissions? A comparative experiment of different on-field straw management practices. *Agriculture, Ecosystems & Environment* **239**, 143-153 (2017).
62 Shin, S. R., Im, S., Mostafa, A., Lee, M. K., Yun, Y. M., Oh, S. E., & Kim, D. H. . Effects of pig slurry acidification on methane emissions during storage and subsequent biogas production. *Water research* **152**, 234-240 (2019).
63 Wassman, R. *et al.* Characterization of Methane Emissions from Rice Fields in Asia. III. Mitigation Options and Future Research Needs. *Nutrient Cycling in Agroecosystems* **58**, 23–36 (2000). https://doi.org:doi:10.1023/A:1009874014903
64 Nguyen, H. V. *et al.* Energy efficiency, greenhouse gas emissions, and cost of rice strawcollection in the mekong river delta of vietnam. *Field Crops Research* **198** 16–22 (2016).
65 Kaur, J., & Singh, A. . Direct Seeded Rice: Prospects, Problems/Constraints and Researchable Issues in India. *Current agriculture research Journal* **5**, 13 (2017).
66 Ramesh, T., & Rathika, S. Evaluation of rice cultivation systems for greenhouse gases emission and productivity. *Int. J. Ecol. Environ. Sci* **2**, 49-54 (2020).
67 Susilawati, H. L., Setyanto, P., Kartikawati, R., & Sutriadi, M. T. The opportunity of direct seeding to mitigate greenhouse gas emission from paddy rice field. *IOP Conference Series: Earth and environmental Science* **393**, 012042 (2019).
68 Cisneros de la Cueva, S., Balagurusamy, N., Pérez-Vega, S., Pérez-Reyes, I., Vázquez-Castillo, J., Zavala Díaz de la Serna, F., Salmerón-Ochoa, I. . Effects of different nitrogen sources on methane production, free ammonium and hydrogen sulfide in anaerobic digestion of cheese whey with cow manure. *Revista Mexicana De Ingeniería Química* **20** (2021). https://doi.org:https://doi.org/10.24275/rmiq/Bio2566
69 da Silva Cardoso, A., Quintana, B. G., Janusckiewicz, E. R., de Figueiredo Brito, L., da Silva Morgado, E., Reis, R. A., & Ruggieri, A. C. . How do methane rates vary with soil moisture and compaction, N compound and rate, and dung addition in a tropical soil? *International journal of biometeorology* **63**, 1533-1540 (2019).
70 Linquist, B. A., Adviento-Borbe, M. A., Pittelkow, C. M., Kessel, C. v. & Groenigen, K. J. v. Fertilizer management practices and greenhouse gas emissions from rice systems: A quantitative review and analysis. *Field Crops Research* **135**, 10–21 (2012).

71 Luo, Y., Li, G., Luo, W., Schuchardt, F., Jiang, T., & Xu, D. Effect of phosphogypsum and dicyandiamide as additives on NH3, N2O and CH4 emissions during composting. *Journal of environmental sciences* **25**, 1338-1345 (2013).

72 Yang, F., Li, G., Shi, H., & Wang, Y. . Effects of phosphogypsum and superphosphate on compost maturity and gaseous emissions during kitchen waste composting. . *Waste management* **36** (2015).

73 Yuan, J., Li, Y., Chen, S., Li, D., Tang, H., Chadwick, D., ... & Li, G. Effects of phosphogypsum, superphosphate, and dicyandiamide on gaseous emission and compost quality during sewage sludge composting. *Bioresource technology* **270**, 368-376 (2018).

74 Feng, J. *et al.* Impacts of cropping practices on yield-scaled greenhouse gas emissions from rice fields in China: A meta-analysis. *Agriculture, Ecosystems and Environment* **164**, 220– 228 (2013).

75 Jiao, Z. *et al.* Water Management Influencing Methane and Nitrous Oxide Emissions from Rice Field in Relation to Soil Redox and Microbial Community. *Communications in Soil Science and Plant Analysis* **37** (2006).

76 Tariq, A. *et al.* Mitigating CH4 and N2O emissions from intensive rice production systems in northern Vietnam: Efficiency of drainage patterns in combination with rice residue incorporation. *Agriculture, Ecosystems and Environment* **249**, 101–111 (2017).

77 Towprayoon, S., Smakgahn, K. & Poonkaew, S. Mitigation of methane and nitrous oxide emissions from drained irrigated rice fields. *Chemosphere* **59**, 1547–1556 (2005). https://doi.org:doi:10.1016/j.chemosphere.2005.02.009

78 Thu, T. N., Phuong, L. B. T., Van, T. M. & Hong, S. N. Effect of Water Regimes and Organic Matter Strategies on Mitigating Green House Gas Emission from Rice Cultivation and Co-benefits in Agriculture in Vietnam. *International Journal of Environmental Science and Development* **7** (2016).

79 Tyagi, L., Kumari, B. & Singh, S. N. Water management — A tool for methane mitigation from irrigated paddy fields. *Science of the Total Environment* **408**, 1085–1090 (2010).

80 Yang, S., Peng, S., Xu, J., Luo, Y. & Li, D. Methane and nitrous oxide emissions from paddy field as affected by water-saving irrigation. *Physics and Chemistry of the Earth, Parts A/B/C* **53-54**, 30-37 (2012).

81 Yue, J. *et al.* Methane and nitrous oxide emissions from rice field and related microorganism in black soil, northeastern China. *Nutrient Cycling in Agroecosystems* **73**, 293–301 (2005).

82 Nalley, L., Bruce Linquist, Kent Kovacs & Anders, M. The Economic Viability of Alternative Wetting and Drying Irrigation in Arkansas Rice Production. *Crop Economics, Production & Management* **105**, 579–587 (2015).

83 Yu, K., Chen, G. & Jr, W. H. P. Reduction of global warming potential contribution from a rice field by irrigation, organic matter, and fertilizer management. *Global Biogeochemical cycles* **18** (2004).

84 Chidthaisong, A. *et al.* Evaluating the effects of alternate wetting and drying (AWD) on methane and nitrous oxide emissions from a paddy field in Thailand. *Soil Science and Plant Nutrition* **64**, 31-38 (2017). https://doi.org:10.1080/00380768.2017.1399044

85 LaHue, G. T., Chaney, R. L., Adviento-Borbe, M. A. & Linquist, B. A. Alternate wetting and drying in high yielding direct-seeded rice systems accomplishes multiple environmental and agronomic objectives. *Agriculture, Ecosystems & Environment* **229**, 30-39 (2016). https://doi.org:10.1016/j.agee.2016.05.020

86 Oo, A. Z. *et al.* Mitigation Potential and Yield-Scaled Global Warming Potential of Early-Season Drainage from a Rice Paddy in Tamil Nadu, India. *Agronomy* **8** (2018). https://doi.org:10.3390/agronomy8100202

87 Runkle, B. R. K. *et al.* Methane Emission Reductions from the Alternate Wetting and Drying of Rice Fields Detected Using the Eddy Covariance Method. *Environ Sci Technol* **53**, 671-681 (2019). https://doi.org:10.1021/acs.est.8b05535

88 Setyanto, P. *et al.* Alternate wetting and drying reduces methane emission from a rice paddy in Central Java, Indonesia without yield loss. *Soil Science and Plant Nutrition* **64**, 23-30 (2017). https://doi.org:10.1080/00380768.2017.1409600

89 Sriphirom, P., Chidthaisong, A., Yagi, K., Tripetchkul, S. & Towprayoon, S. Evaluation of biochar applications combined with alternate wetting and drying (AWD) water management in rice field as a methane mitigation option for farmers' adoption. *Soil Science and Plant Nutrition* **66**, 235-246 (2019). https://doi.org:10.1080/00380768.2019.1706431

90 Tirol-Padre, A., Minamikawa, K., Tokida, T., Wassmann, R. & Yagi, K. Site-specific feasibility of alternate wetting and drying as a greenhouse gas mitigation option in irrigated rice fields in Southeast Asia: a synthesis. *Soil Science and Plant Nutrition* **64**, 2-13 (2017). https://doi.org:10.1080/00380768.2017.1409602

91 Tran, D. H., Hoang, T. N., Tokida, T., Tirol-Padre, A. & Minamikawa, K. Impacts of alternate wetting and drying on greenhouse gas emission from paddy field in Central Vietnam. *Soil Science and Plant Nutrition* **64**, 14-22 (2017). https://doi.org:10.1080/00380768.2017.1409601

92 Habtewold, J., Gordon, R., Sokolov, V., VanderZaag, A., Wagner-Riddle, C., & Dunfield, K. Reduction in Methane Emissions From Acidified Dairy Slurry Is Related to Inhibition of Methanosarcina Species. *Frontiers in microbiology* **9**, 2086 (2018).

93 Sommer, S. G., Clough, T. J., Balaine, N., Hafner, S. D., & Cameron, K. C. Transformation of Organic Matter and the Emissions of Methane and Ammonia during Storage of Liquid Manure as Affected by Acidification. *Journal of environmental quality* **46**, 514-521 (2017).

94 Misselbrook, T. H., Hunt, J., Perazzolo, F., & Provolo, G. Greenhouse gas and ammonia emissions from slurry storage: Impacts of temperature and potential mitigation through covering (pig slurry) or acidification (cattle slurry). *Journal of Environmental Quality* **45**, 1520-1530 (2016).

95 Kavanagh, I., Burchill, W., Healy, M. G., Fenton, O., Krol, D. J., & Lanigan, G. J. Mitigation of ammonia and greenhouse gas emissions from stored cattle slurry using acidifiers and chemical amendments. *Journal of Cleaner Production* **237**, 117822 (2019).

96 Petersen, S. O., Andersen, A. J. & Eriksen, J. Effects of Cattle Slurry Acidification on Ammonia and Methane Evolution during Storage. *Journal of Environmental Quality Abstract - Atmospheric Pollutants and Trace Gases* **41**, 88-94 (2012).

97 Holly, M. A., Larson, R. A., Powell, J. M., Ruark, M. D., & Aguirre-Villegas, H. . Greenhouse gas and ammonia emissions from digested and separated dairy manure during storage and after land application. *Agriculture, Ecosystems & Environment* **239**, 410-419 (2017).

98 VanderZaag, A. C., Baldé, H., Crolla, A., Gordon, R. J., Ngwabie, N. M., Wagner-Riddle, C., ... & MacDonald, J. D. Potential methane emission reductions for two manure treatment technologies. *Environmental technology* **39**, 851-858 (2018).

99 Oshita, K., Okumura, T., Takaoka, M., Fujimori, T., Appels, L., & Dewil, R. Methane and nitrous oxide emissions following anaerobic digestion of sludge in Japanese sewage treatment facilities. *Bioresource technology* **171**, 175-181 (2014).

100 Massé, D. I., Jarret, G., Hassanat, F., Benchaar, C., & Saady, N. M. C. Effect of increasing levels of corn silage in an alfalfa-based dairy cow diet and of manure management practices on manure fugitive methane emissions. *Agriculture, Ecosystems & Environment* **221**, 109-114 (2016).

101 Le Riche, E. L., VanderZaag, A. C., Wagner-Riddle, C., Dunfield, K., Sokolov, V. K., & Gordon, R. . Do volatile solids from bedding materials increase greenhouse gas emissions for stored dairy manure? *Canadian Journal of Soil Science* **97**, 512-521 (2017).

102 Van der Heyden, C., Demeyer, P., & Volcke, E. I. . Mitigating emissions from pig and poultry housing facilities through air scrubbers and biofilters: State-of-the-art and perspectives. *Biosystems Engineering* **134**, 74-93 (2015).

103 Laguë, C., Gaudet, É., Agnew, J., & Fonstad, T. A. . Greenhouse gas and odor emissions from liquid swine manure storage facilities in Saskatchewan. *American Society of Agricultural and Biological Engineers.*, 1 (2004).
104 Chiumenti, A., da Borso, F., Pezzuolo, A., Sartori, L., & Chiumenti, R. Ammonia and greenhouse gas emissions from slatted dairy barn floors cleaned by robotic scrapers. *Research in Agricultural Engineering* **64**, 26-33 (2018).
105 Sommer, S. G., Petersen, S. O., & Møller, H. B. Algorithms for calculating methane and nitrous oxide emissions from manure management. *Nutrient Cycling in Agroecosystems* **69**, 143-154 (2004).
106 Ma, S., Sun, X., Fang, C., He, X., Han, L., & Huang, G. . Exploring the mechanisms of decreased methane during pig manure and wheat straw aerobic composting covered with a semi-permeable membrane. *Waste management* **78**, 393-400 (2018).
107 Gilsanz, C., Báez, D., Misselbrook, T. H., Dhanoa, M. S., & Cárdenas, L. M. Development of emission factors and efficiency of two nitrification inhibitors, DCD and DMPP. *Agriculture, Ecosystems & Environment* **216**, 1-8 (2016).
108 Volpi, I., Laville, P., Bonari, E., o di Nasso, N. N., & Bosco, S. Improving the management of mineral fertilizers for nitrous oxide mitigation: The effect of nitrogen fertilizer type, urease and nitrification inhibitors in two different textured soils. *Geoderma* **307**, 181-188 (2017).
109 Guardia, G., Marsden, K. A., Vallejo, A., Jones, D. L., & Chadwick, D. R. Determining the influence of environmental and edaphic factors on the fate of the nitrification inhibitors DCD and DMPP in soil. *Science of the Total Environment* **624**, 1202-1212 (2018).
110 Xia, L., Lam, S. K., Chen, D., Wang, J., Tang, Q., & Yan, X. Can knowledge-based N management produce more staple grain with lower greenhouse gas emission and reactive nitrogen pollution? A meta-analysis. *Global Change Biology* **23**, 1917-1925 (2017).
111 Luo, Z., Lam, S. K., Fu, H., Hu, S., & Chen, D. Temporal and spatial evolution of nitrous oxide emissions in China: Assessment, strategy and recommendation. *Journal of Cleaner Production* **223**, 360-367 (2019).
112 Gao, J., Luo, J., Lindsey, S., Shi, Y., Sun, Z., Wei, Z., & Wang, L. Benefits and Risks for the Environment and Crop Production with Application of Nitrification Inhibitors in China. *Journal of soil science and plant nutrition* **21**, 497-512 (2021).
113 Akiyama, H., Yan, X. & Yagi, K. Evaluation of effectiveness of enhanced-efficiency fertilizers as mitigation options for N2O and NO emissions from agricultural soils: meta-analysis. *Global Change Biology* **16**, 1837–1846 (2010).
114 Bates, J., Brophy, N., Harfoot, M. & Webb, J. Sectoral Emission Reduction Potentials and Economic Costs for Climate Change (SERPEC-CC) Agriculture: methane and nitrous oxide. (2009).
115 Wu, D. *et al.* Nitrification inhibitors mitigate N 2 O emissions more effectively under straw-induced conditions favoring denitrification. *Soil Biology and Biochemistry* **104**, 197-207 (2017). https://doi.org:10.1016/j.soilbio.2016.10.022
116 Zhu, K., Bruun, S. & Jensen, L. S. Nitrogen transformations in and N2O emissions from soil amended with manure solids and nitrification inhibitor. *European Journal of Soil Science* **67**, 792-803 (2016). https://doi.org:10.1111/ejss.12385
117 Torralbo, F. *et al.* Dimethyl pyrazol-based nitrification inhibitors effect on nitrifying and denitrifying bacteria to mitigate N2O emission. *Sci Rep* **7**, 13810 (2017). https://doi.org:10.1038/s41598-017-14225-y
118 Duncan, E. W., Dell, C. J., Kleinman, P. J. A., & Beegle, D. B. Nitrous Oxide and Ammonia Emissions from Injected and Broadcast-Applied Dairy Slurry. *Journal of Environmental Quality* **46**, 36-44 (2017).
119 Sadeghpour, A., Ketterings, Q. M., Vermeylen, F., Godwin, G. S., & Czymmek, K. J. . Nitrous Oxide Emissions from Surface versus Injected Manure in Perennial Hay Crops. *Soil Science Society of America Journal* **82**, 156-166 (2018).

120 Hunt, D., Bittman, S., Chantigny, M., & Lemke, R. Year-Round N2O Emissions From Long-Term Applications of Whole and Separated Liquid Dairy Slurry on a Perennial Grass Sward and Strategies for Mitigation. *Frontiers in Sustainable Food Systems* **3**, 86 (2019).
121 Eagle, A. J. *et al.* Technical Working Group on agricultural Greenhouse Gases (T-AGG) REPORT Greenhouse Gas Mitigation Potential of Agricultural Land Management in the United States A Synthesis of the Literature. (2012).
122 Moran, D. *et al.* UK marginal cost curves for the agriculture, forestry, land-use and land-use change sector out to 2022 and to provide scenario analysis for possible abatement options out to 2050 –RMP4950.Defra. (2008).
123 Deng, J., Guo, L., Salas, W., Ingraham, P., Charrier-Klobas, J. G., Frolking, S., & Li, C. . Changes in Irrigation Practices Likely Mitigate Nitrous Oxide Emissions From California Cropland. *Global Biogeochemical cycles* **32**, 1514-1527 (2018).
124 Kuang, W., Gao, X., Tenuta, M., & Zeng, F. A global meta-analysis of nitrous oxide emission from drip-irrigated cropping system. *Glob Change Biology* **27**, 3244-3256 (2021).
125 Wang, G., Liang, Y., Zhang, Q., Jha, S. K., Gao, Y., Shen, X., ... & Duan, A. . Mitigated CH4 and N2O emissions and improved irrigation water use efficiency in winter wheat field with surface drip irrigation in the North China Plain. *Agricultural Water Management* **163**, 403-307 (2016).
126 Sanchez-Martín, L., Meijide, A., Garcia-Torres, L., & Vallejo, A. Combination of drip irrigation and organic fertilizer for mitigating emissions of nitrogen oxides in semiarid climate. *Agriculture, Ecosystems & Environment* **137**, 99-107 (2010).
127 Borchard, N., Schirrmann, M., Cayuela, M. L., Kammann, C., Wrage-Mönnig, N., Estavillo, J. M., ... & Novak, J. Biochar, soil and land-use interactions that reduce nitrate leaching and N2O emissions: A meta-analysis. *Science of the Total Environment* **651**, 2354-2364 (2019).
128 Dawar, K., Fahad, S., Alam, S. S., Khan, S. A., Dawar, A., Younis, U., ... & Dick, R. P. Infuence of variable biochar concentration on yield-scaled nitrous oxide emissions, Wheat yield and nitrogen use efciency. *Scientific Reports* **11**, 1-10 (2021).
129 Liu, Q., Zhang, Y., Liu, B., Amonette, J. E., Lin, Z., Liu, G., ... & Xie, Z. How does biochar influence soil N cycle? A meta-analysis. *Plant and soil* **426**, 211-225 (2018).
130 Puga, A. P., Queiroz, M. C. D. A., Ligo, M. A. V., Carvalho, C. S., Pires, A. M. M., Marcatto, J. D. O. S., & Andrade, C. A. D. . Nitrogen availability and ammonia volatilization in biochar-based fertilizers. *Archives of agronomy and soil science* **66**, 992-1004 (2020).
131 Cao, Q., Miao, Y., Feng, G., Gao, X., Liu, B., Liu, Y., ... & Zhang, F. . Improving nitrogen use efficiency with minimal environmental risks using an active canopy sensor in a wheat-maize cropping system. *Field Crops Research* **214**, 365-372 (2017).
132 Song, X., Liu, M., Ju, X., Gao, B., Su, F., Chen, X., & Rees, R. M. Nitrous Oxide Emissions Increase Exponentially When Optimum Nitrogen Fertilizer Rates Are Exceeded in the North China Plain. *Environmental Science & Technology* **52**, 12504-12513 (2018).
133 Winiwarter, W., Höglund-Isaksson, L., Klimont, Z., Schöpp, W. & Amann, M. Technical opportunities to reduce global anthropogenic emissions of nitrous oxide. *Environmental Research Letters* **13**, 014011 (2018). https://doi.org:10.1088/1748-9326/aa9ec9
134 Drury, C. F., Reynolds, W. D., Yang, X., McLaughlin, N. B., Calder, W., & Phillips, L. A. . Diverse rotations impact microbial processes, seasonality and overall nitrous oxide emissions from soils. *Soil Science Society of America Journal* **85**, 1448-1464 (2021).
135 Mahama, G. Y., Prasad, P. V. V., Roozeboom, K. L., Nippert, J. B., & Rice, C. W. Reduction of Nitrogen Fertilizer Requirements and Nitrous Oxide Emissions Using Legume Cover Crops in a No-Tillage Sorghum Production System. *Sustainability* **12**, 4403 (2020).
136 Behnke, G. D., Zuber, S. M., Pittelkow, C. M., Nafziger, E. D., & Villamil, M. B. Long-term crop rotation and tillage effects on soil greenhouse gas emissions and crop production in Illinois, USA. *Agriculture, Ecosystems & Environment* **261**, 62-70 (2018).
137 Behnke, G. D., & Villamil, M. B. Cover crop rotations affect greenhouse gas emissions and crop production in Illinois, USA. *Field Crops Research* **241**, 107580 (2019).

138 Abagandura, G. O., Şentürklü, S., Singh, N., Kumar, S., Landblom, D. G., & Ringwall, K. Impacts of crop rotational diversity and grazing under integrated crop-livestock system on soil surface greenhouse gas fluxes. *Plos one* **14**, e0217069 (2019).
139 Wegner, B. R., Chalise, K. S., Singh, S., Lai, L., Abagandura, G. O., Kumar, S., ... & Jagadamma, S. Response of Soil Surface Greenhouse Gas Fluxes to Crop Residue Removal and Cover Crops under a Corn–Soybean Rotation. *Journal of Environmental Quality* **47**, 1146-1154 (2018).
140 Weiler, D. A., Tornquist, C. G., Parton, W., dos Santos, H. P., Santi, A., & Bayer, C. Crop Biomass, Soil Carbon, and Nitrous Oxide as Affected by Management and Climate: A DayCent Application in Brazil. *Soil Science Society of America Journal* **81**, 945-955 (2017).
141 Van Kessel, C. *et al.* Climate, duration, and N placement determine N2O emissions in reduced tillage systems: a meta-analysis. *Global Change Biology* **19**, 33–44 (2013).
142 Congreves, K. A., Brown, S. E., Németh, D. D., Dunfield, K. E., & Wagner-Riddle, C. . Differences in field-scale N2O flux linked to crop residue removal under two tillage systems in cold climates. *Gcb bioenergy* **9**, 555-680 (2017).
143 Machado, P. V. F., Farrell, R. E., Bell, G., Taveira, C. J., Congreves, K. A., Voroney, R. P., ... & Wagner-Riddle, C. Crop residues contribute minimally to spring-thaw nitrous oxide emissions under contrasting tillage and crop rotations. *Biology and biochemistry* **152**, 108057 (2021).
144 Fiorini, A., Maris, S. C., Abalos, D., Amaducci, S., & Tabaglio, V. Combining no-till with rye (Secale cereale L.) cover crop mitigates nitrous oxide emissions without decreasing yield. *Soil and Tillage Research* **196**, 104442 (2020).
145 Lala, A. O., Oso, A. O., Osafo, E. L., & Houdijk, J. G. . Impact of reduced dietary crude protein levels and phytase enzyme supplementation on growth response, slurry characteristics, and gas emissions of growing pigs. *Animal Science Journal* **91**, e13381 (2020).
146 Trabue, S. L., Kerr, B. J., Scoggin, K. D., Andersen, D., & Van Weelden, M. Swine diets impact manure characteristics and gas emissions: Part I protein level. *Science of the Total Environment* **755**, 142528 (2021).
147 Bao, Y., Zhou, K., & Zhao, G. Nitrous oxide emissions from the urine of beef cattle as regulated by dietary crude protein and gallic acid. *Journal of animal science* **96**, 3699-3711 (2018).
148 Li, Q. F., Trottier, N., & Powers, W. . Feeding reduced crude protein diets with crystalline amino acids supplementation reduce air gas emissions from housing. *Journal of animal science* **93**, 721-730 (2015).
149 Zhou, K., Bao, Y., & Zhao, G. . Effects of dietary crude protein and tannic acid on nitrogen excretion, urinary nitrogenous composition and urine nitrous oxide emissions in beef cattle. *Journal of animal physiology and animal nutrition* **103**, 1675-1683 (2019).
150 Baral, K. R., Labouriau, R., Olesen, J. E., & Petersen, S. O. Nitrous oxide emissions and nitrogen use efficiency of manure and digestates applied to spring barley. *Agriculture, Ecosystems & Environment* **239**, 188-198 (2017).
151 Grave, R. A., da Silveira Nicoloso, R., Cassol, P. C., da Silva, M. L. B., Mezzari, M. P., Aita, C., & Wuaden, C. R. . Determining the effects of tillage and nitrogen sources on soil N2O emission. *Soil and Tillage Research* **175**, 1-12 (2018).
152 Owusu-Twum, M. Y., Loick, N., Cardenas, L. M., Coutinho, J., Trindade, H., & Fangueiro, D. . Nitrogen dynamics in soils amended with slurry treated by acid or DMPP addition. *Biology and Fertility of Soils* **53**, 339-347 (2017).
153 Petersen, S. O., Højberg, O., Poulsen, M., Schwab, C., & Eriksen, J. . Methanogenic community changes, and emissions of methane and other gases, during storage of acidified and untreated pig slurry. *Journal of applied microbiology* **117**, 160-172 (2014).
154 Emmerling, C., Krein, A., & Junk, J. Meta-Analysis of Strategies to Reduce NH3 Emissions from Slurries in European Agriculture and Consequences for Greenhouse Gas Emissions. *Agronomy* **10**, 1633 (2020).

155 Park, S. H., Lee, B. R., Jung, K. H., & Kim, T. H. Acidification of pig slurry effects on ammonia and nitrous oxide emissions, nitrate leaching, and perennial ryegrass regrowth as estimated by 15N-urea flux. *Asian-Australasian journal of animal sciences* **31**, 457 (2018).
156 Fangueiro, D., Ribeiro, H., Coutinho, J., Cardenas, L., Trindade, H., Cunha-Queda, C., ... & Cabral, F. Nitrogen mineralization and CO 2 and N 2 O emissions in a sandy soil amended with original or acidified pig slurries or with the relative fractions. *Biology and Fertility of Soils* **46**, 383-391 (2010).
157 Berg, W., Türk, M., & Hellebrand, H. J. . Effects of Acidifying Liquid Cattle Manure with Nitric or Lactic Acid on Gaseous Emissions *Proceedings Workshop on Agricultural Air Quality: State of the Science*, 492-498 (2006).
158 Ecofys. Development of F-gas module for TIMER model. Authors: Dr. Jochen Harnisch, Sebastian Klaus, Sina Wartmann and Jan-Martin Rhiemeier. Project number: PECSDE082196 Client: Netherlands Environmental Assessment Agency MNP. (2006).
159 Thakur, S. & Solanski, H. Role of Methane in Climate Change and Options for Mitigation-A Brief Review. *International Association of Biologicals and computationoal Digest* **6**, 85-99 (2021).
160 El-Mrabet, R. *et al.* Phosphogypsum amendment effect on radionuclide content in drainage water and marsh soils from southwestern Spain. *Journal of environmental Quality* **32**, 1262-1268 (2003).
161 Papastefanou, C., Stoulos, S., Ioannidou, A. & Manolopoulou, M. The application of phosphogypsum in agriculture and the radiological impact. *Journal of environmental radioactivity* **89**, 188-198 (2006).
162 Abril, J. M. *et al.* The cumulative effect of three decades of phosphogypsum amendments in reclaimed marsh soils from SW Spain: 226Ra, 238U and Cd contents in soils and tomato fruit. *Science of the Total Environment* **403**, 80-88 (2008).
163 Hurtado, M. D., Enamorado, S. M., Andreu, L., Delgado, A. & Abril, J. M. Drain flow and related salt losses as affected by phosphogypsum amendment in reclaimed marsh soils from SW Spain. *Geoderma* **161**, 43-49 (2011).
164 Elloumi, N. *et al.* Effect of phosphogypsum on growth, physiology, and the antioxidative defense system in sunflower seedlings. *Environmental Science and Pollution Research* **22**, 14829-14840 (2015).
165 Peng, X. *et al.* The addition of biochar as a fertilizer supplement for the attenuation of potentially toxic elements in phosphogypsum-amended soil. *Journal of Cleaner Production* **277**, 124052 (2020).
166 Rahman, M. M. Potential benefits of dry direct seeded rice culture: A review. *Fundamental and Applied Agriculture* **4**, 744-758 (2019).
167 Gullett, B. & Touati, A. PCDD/F emissions from burning wheat and rice field residue. *Atmospheric Environment* **37**, 4893-4899 (2003).
168 ARAI, T. *et al.* Bronchial asthma induced by rice. *Internal medicine* **37**, 98-101 (1998).
169 Lin, L. F., Lee, W. J., Li, H. W., Wang, M. S. & Chang-Chien, G. P. Characterization and inventory of PCDD/F emissions from coal-fired power plants and other sources in Taiwan. *Chemosphere* **68**, 1642-1649 (2007).
170 Tipayarom, D. & Oanh, N. K. Effects from open rice straw burning emission on air quality in the Bangkok Metropolitan Region. . *Science Asia* **33**, 339-345 (2007).
171 Torigoe, K. *et al.* Influence of emission from rice straw burning on bronchial asthma in children. *Pediatrics International* **42**, 143-150 (2000).
172 Kanokkanjana, K., Cheewaphongphan, P. & Garivait, S. Black carbon emission from paddy field open burning in Thailand. . *IPCBEE Proc* **6** (2011).
173 Zucconi, F., Pera, A., Forte, M. & De Bertolid, M. Evaluating toxicity of immature compost. *Biocycle* **22**, 54-57 (1981).

174 Kaur, P., Kocher, G. S. & Taggar, M. S. Enhanced bio-composting of rice straw using agribultural residues: an alternate to burning. *International Journal of Recycling of Organic Waste in Agriculture* **8**, 479-483 (2019).
175 Wang, W. *et al.* Mitigating effects of ex situ application of rice straw on CH4 and N2O emissions from paddy-upland coexisting system. . *Scientific Reports* **6**, 1-8 (2016).
176 Chhabra, A., Manjunath, K. R., Panigrahy, S. & Parihar, J. Spatial Pattern of Methane Emissions from Indian Livestock. *Current Science* **96**, 683–689 (2009).
177 Lay, J. J., Li, Y. Y. & Noike, T. Influences of pH and moisture content on the methane production in high-solids sludge digestion. *Water Research* **31**, 1518-1524 (1997).
178 Pind, P. F., Angelidaki, L., Ahring, B. K., Stamatelatou, K. & Lyberatos, G. Monitoring and control of anaerobic reactors. *Biomethanation II*, 135-182 (2003).
179 Angelidaki, L., Ellegaard, L. & Ahring, B. K. Applications of the anaerobic digestion process. *Biomethanation II*, 1-33 (2003).
180 Angelidaki, L., Karakashev, D., Batstone, D. J., Plugge, C. M. & Stams, A. J. Biomethanation and its potential. *Methods in enzymology* **494**, 327-351 (2011).
181 Christensen, M. L., Christensen, K. V. & S.G., S. Solid-liquid separation of animal slurry. *Animal manure recycling: treatment and management. Wiley, New York*, 105-130 (2013).
182 Fangueiro, D., Senbayran, M., Trindade, H. & Chadwick, D. Cattle slurry treatment by screw press separation and chemically enhanced settling: effect on greenhouse gas emissions after land spreading and grass yield. . *Bioresource Technology* **99**, 7132-7142 (2008).
183 Stockmann, U. *et al.* The knowns, known unknowns and unknowns of sequestration of soil organic carbon. *Agriculture, Ecosystems & Environment* **164**, 80-99 (2013). https://doi.org:10.1016/j.agee.2012.10.001
184 Karakurt, I., Aydin, G. & Aydiner, K. Sources and mitigation of methane emissions by sectors: A critical review. *Renewable Energy* **39**, 40-48 (2012). https://doi.org:10.1016/j.renene.2011.09.006
185 Hinde, B. P., Mitchell, I. & Riddell, M. COMETTM - A New Ventilation Air Methane (VAM) Abatement Technology. *Johnson Matthey Technology Review* **60**, 211-221 (2016). https://doi.org:10.1595/205651316x692059
186 Hui, K. S., Kwong, C. W. & Chao, C. Y. H. Methane emission abatement by Pd-ion-exchanged zeolite 13X with ozone. *Energy & Environmental Science* **3**, 1092 (2010). https://doi.org:10.1039/c002669g
187 Lebrero, R. *et al.* Exploring the potential of fungi for methane abatement: Performance evaluation of a fungal-bacterial biofilter. *Chemosphere* **144**, 97-106 (2016). https://doi.org:10.1016/j.chemosphere.2015.08.017
188 Patel, S., Tremain, P., Sandford, J., Moghtaderi, B. & Shah, K. Empirical Kinetic Model of a Stone Dust Looping Carbonator for Ventilation Air Methane Abatement. *Energy & Fuels* **30**, 1869-1878 (2016). https://doi.org:10.1021/acs.energyfuels.5b02206
189 Yusuf, R. O., Noor, Z. Z., Abba, A. H., Hassan, M. A. A. & Din, M. F. M. Methane emission by sectors: A comprehensive review of emission sources and mitigation methods. *Renewable and Sustainable Energy Reviews* **16**, 5059-5070 (2012). https://doi.org:10.1016/j.rser.2012.04.008
190 Bylin, C. *et al.* Designing the Ideal Offshore Platform Methane Mitigation Strategy. (US Environmental Protection Agency, SPE, 2010).
191 Lipsky, R. Gas-to-Liquids Technology Offers Solution for Stranded Gas. The American oil and gas reported. (2014).
192 Lechtenböhmer, S. & Dienst, C. Future development of the upstream greenhouse gas emissions from natural gas industry, focussing on Russian gas fields and export pipelines. *Journal of Integrative Environmental Sciences* **7**, 39-48 (2010). https://doi.org:10.1080/19438151003774463

193 Ravikumar, A. P. & Brandt, A. R. Designing better methane mitigation policies: the challenge of distributed small sources in the natural gas sector. *Environmental Research Letters* **12**, 044023 (2017). https://doi.org:10.1088/1748-9326/aa6791
194 Lopez, J. C. *et al.* Biotechnologies for greenhouse gases (CH(4), N(2)O, and CO(2)) abatement: state of the art and challenges. *Appl Microbiol Biotechnol* **97**, 2277-2303 (2013). https://doi.org:10.1007/s00253-013-4734-z
195 Barcon, T., Hernandez, J., Gomez-Cuervo, S., Garrido, J. M. & Omil, F. Characterization and biological abatement of diffuse methane emissions and odour in an innovative wastewater treatment plant. *Environ Technol* **36**, 2105-2114 (2015). https://doi.org:10.1080/09593330.2015.1021859
196 Reid, M. C., Guan, K., Wagner, F. & Mauzerall, D. L. Global methane emissions from pit latrines. *Environ Sci Technol* **48**, 8727-8734 (2014). https://doi.org:10.1021/es501549h
197 Hoekman, S. K. Review of Nitrous Oxide (N2O) Emissions from Motor Vehicles. *SAE International Journal of Fuels and Lubricants* **13.1**, 79-98 (2020).
198 Toyoda, S. Y., S.; Arai, S.; Nara, H.; Yoshida, N.; Kashiwakura, K.; Akiyama, K. Isotopomeric characterization of N2O produced, consumed, and emitted by automobiles. . *Rapid Commun. Mass Spectrom* **22**, 603-612 (2008).
199 Dasch, J. M. Nitrous oxide emissions from Vehicles. *J. Air Waste Manage. Assoc* **42**, 63-67 (1992).
200 Eom, W.-H., Ayoub, M. & Yoo, K.-S. Catalytic Decomposition of N2O at Low Temperature by Reduced Cobalt Oxides. *Journal of Nanoscience and Nanotechnology* **16**, 4647-4654 (2016). https://doi.org:10.1166/jnn.2016.11026
201 Harnisch, J. *et al.* IPCC Guidelines for National Greenhouse Gas Inventories, Chapter 3 Chemical Industry. (2006).
202 Nunotani, N., Nagai, R. & Imanaka, N. Direct catalytic decomposition of nitrous oxide gas over rhodium supported on lanthanum silicate. *Catalysis Communications* **87**, 53-56 (2016). https://doi.org:10.1016/j.catcom.2016.08.032
203 Zhang, R., Hua, C., Wang, B. & Jiang, Y. N2O Decomposition over Cu–Zn/γ–Al2O3 Catalysts. *Catalysts* **6**, 200 (2016). https://doi.org:10.3390/catal6120200
204 Li, L., Xu, J., Hu, J. & Han, J. Reducing nitrous oxide emissions to mitigate climate change and protect the ozone layer. *Environ Sci Technol* **48**, 5290-5297 (2014). https://doi.org:10.1021/es404728s
205 Isupova, L. A. & Ivanova, Y. A. Removal of nitrous oxide in nitric acid production. *Inetics and Catalysis* **60**, 744-760 (2019).
206 Han, D., Zhao, Y., Xue, B. & Chai, X. Effect of bio-column composed of aged refuse on methane abatement – A novel configuration of biological oxidation in refuse landfill. *Journal of Environmental Sciences* **22**, 769-776 (2010). https://doi.org:10.1016/s1001-0742(09)60175-3
207 Park, S., Lee, C.-H., Ryu, C.-R. & Sung, K. Biofiltration for Reducing Methane Emissions from Modern Sanitary Landfills at the Low Methane Generation Stage. *Water, Air, and Soil Pollution* **196**, 19-27 (2008). https://doi.org:10.1007/s11270-008-9754-4
208 Widory, D., Proust, E., Bellenfant, G. & Bour, O. Assessing methane oxidation under landfill covers and its contribution to the above atmospheric CO2 levels: The added value of the isotope (δ13C and δ18O CO2; δ13C and δD CH4) approach. *Waste Management* **32**, 1685 - 1692 (2012).
209 Abichou, T., Kormi, T., Marsh, A. & Wang, C. Phytocaps for Landfill Emission Reduction in Australia. Geo-Chicago 2016: Sustainable Geoenvironmental Systems. Conference paper., 222-231 (2016). https://doi.org:10.1061/9780784480144.022
210 Rose, J. L., Mahler, C. F. & Izzo, R. L. D. S. Comparison of the methane oxidation rate in four medai. *Revista Brasileira de Ciência do Solo* **36**, 803-812 (2012).
211 IPCC/TEAP. *IPCC/TEAP special report on safeguarding the ozone layer and the global climate system : issues related to hydrofluorocarbons and perfluorocarbons : prepared by Working*

Groups I and III of the Intergovernmental Panel on Climate Change and the Technical and Economic Assessment Panel. (Cambridge University Press, 2005).

212 Kassam, A., Friedrich, T. & Derpsch, R. Successful Experiences and Lessons from Conservation Agriculture Worldwide. *Agronomy* **12** (2022). https://doi.org:10.3390/agronomy12040769

213 OECD. *Trends and Drivers of Agri-environmental Performance in OECD Countries, OECD Publishing, Paris*. (2019).

214 Henderson, B. B. *et al.* Greenhouse gas mitigation potential of the world's grazing lands: Modeling soil carbon and nitrogen fluxes of mitigation practices. *Agriculture, Ecosystems and Environment* **207**, 91-100 (2015).

215 McKinsey. Impact of the financial crisis on carbon economics. Version 2.1 of the Global Greenhouse Gas Abatement Cost Curve. McKinsey&Company. (2010).

216 Jacobsen, B. Costs of slurry separation technologies and alternative use of the solid fraction for biogas production or burning–a Danish perspective. *International Journal of Agricultural Management* **1**, 1-22 (2011).

217 Aguirre-Villegas, H. A. & Larson, R. A. Evaluating greenhouse gas emissions from dairy manure management practices using survey data and lifecycle tools. *Journal of Cleaner Production* **143**, 169-179 (2017).

218 Weiske, A. & Michel, J. Greenhouse gas emissions and mitigation costs of selected mitigation measures in agricultural production. MEACAP WP3 D15a. (2007).

219 Smith, P. *et al.* Greenhouse gas mitigation in agriculture. Philosophical Transactions of the Royal Society of London. *Biological Sciences* **363**, 789–813 (2008).

220 Basak, R. Benefits and costs of nitrogen fertilizer management for climate change mitigation Focus on India and Mexico. CGIAR Research Program on Climate Change, Agriculture and Food Security (CCAFS) Working Paper No. 161. (2015).

221 Maris, S. C., Teira_Esmatges, M. R., Arbonés, A. & Rufat, J. Effect of irrigation, nitrogen application, and a nitrification inhibitor on nitrous oxide, carbon dioxide and methane emissions from an olive (Olea europaea L.) orchard. *Science of total Environment* **538**, 966-978 (2015).

222 Xu, X., Zhang, B., Liu, Y., Xue, Y. & Di, B. Carbon footprints of rice production in five typical rice districts in China. *Acta Ecologica Sinica* **33**, 277-232 (2013).

223 Baccour, S., Albiac, J. & Kahil, T. Cost-Effective Mitigation of Greenhouse Gas Emissions in the Agriculture of Aragon, Spain. *Int J Environ Res Public Health* **18** (2021). https://doi.org:10.3390/ijerph18031084

224 IPCC. Climate Change 2022: Mitigation of Climate Change. Contribution of Working Group III to the Sixth Assessment Report of the Intergovernmental Panel on Climate Change [P.R. Shukla, J. Skea, R. Slade, A. Al Khourdajie, R. van Diemen, D. McCollum, M. Pathak, S. Some, P. Vyas, R. Fradera, M. Belkacemi, A. Hasija, G. Lisboa, S. Luz, J. Malley, (eds.)]. (2022).
