# Supplementary material for: Uncertainty in non-CO2 greenhouse gas mitigation contributes to ambiguity in global climate policy feasibility
Source: Nat Commun. 2023 Jun 2;14:2949. doi: 10.1038/s41467-023-38577-4 (PMC10238505; doi:10.1038/s41467-023-38577-4)
Supplement: Supplementary file 3 — Description of Additional Supplementary Files [file 41467_2023_38577_MOESM3_ESM.pdf]

Description of Additional Supplementary Files

File Name: Supplementary Data 1
Description: CH_4 and N_2O optimistic, default and pessimistic MAC curves as developed and applied in the paper

File Name: Supplementary Software 1
Description: Python-based script that can be used to perform the Monte Carlo analysis to build and analyze the agricultural MACs
