# Supplementary material for: Uncertainty in non-CO2 greenhouse gas mitigation contributes to ambiguity in global climate policy feasibility
Source: Nat Commun. 2023 Jun 2;14:2949. doi: 10.1038/s41467-023-38577-4 (PMC10238505; doi:10.1038/s41467-023-38577-4)
Supplement: Supplementary file 5 — Supplementary Software 1 [file 41467_2023_38577_MOESM5_ESM.zip › Supplementary Software 1/Supplementary Software 1 Legend.docx]

This tool and the assessment of these Non-CO2 Marginal Abatement Cost (MAC) curves are described in the paper, ‘Uncertainty in non-CO2 greenhouse gas mitigation contributes to ambiguity in global climate policy feasibility’ (Harmsen et al., 2023)

This script can be used to generate "optimistic", default & "pessimistic" agriculture non-CO2 MAC curves with high, medium, low reduction potential, respectively. The MACs describe relative reductions (compared to global average values in 2015) at carbon equivalent prices up to 4000 $/tC (in 201 steps) for the 2020-2100 period. The MACs are built up from underlying parameters that describe technical applicability of mitigation measures, reduction efficiencies, implementation barriers, level of overlap between measures, technological progress and costs. In a Monte Carlo analysis, the values for these factors are varied randomly resulting in different (1000x in the current setup) MAC profiles for the various emission sources. The final high, medium, low macs are based on the 5th, 50th and 95th percentile for each time step. There are 5 agricultural sources (CH4: enteric fermentation, rice, manure. N2O: fertilizer, manure). In addition, the tool also generates variants of the enteric fermentation and fertilizer MACs with additional measures added (seaweed and biochar, respectively). These additional (promising, but not fully proven) technologies are included in the optimistic MACs. Note that the tool is not run automatically with FAIR/IMAGE. That's also not needed, since the datafiles that the tool generates are also used as input in FAIR. However, the tool can be run when you want to change or test the underlying MAC parameters. The MC routine includes a seed, meaning that the random values chosen will be saved (for 3 consecutive runs).

The following paragraphs explain the structure of the code below.

#### Constants

This code starts with defining the constants used to calculate the MAC-curves. These constants are given in a certain order, initially, but this is not the order of implementation. This order of implementation is determined on the basis of the costs: the least costly measures are implemented first. This is done later in the code.

The RE-input values are given and written in our initial order. The same goes for the technical applicability. Then, the delta values are given. Then, the implementation potential and technological progress values are given.

Next, the order of implementation is calculated. This order is needed to calculate the correction for overlap values since these depend on previously implemented measures.

The correction for overlap values are a bit more complicated. First, the values for overlap between each of the measures is given. For example: ni1 = {'bc': 0.50, 'irr': 1}, shows the overlap between nitrification inhibitors and biochar and irrigation practices. This is respectively 50% and 100%. The overlap between nitrification inhibitors and spreader maintenance can be found in the line above: spread_m1 = { 'ni': 0.7, 'bc': 1, 'irr': 1}. That overlap is 70%. As a second, we needed to specify for each measure which measures were already implemented. Note here that as you have different countries with different costs and therefore different order of implementation, you will have to specify this for each country that has different order of implementation.

This was written down in an easier way with the order as described on top. This might sound confusing because the order of real implementation is used already to write down which measures were implemented before each measure. However, we use the order on top only so that the order is the same for the different variables and so that we can adjust all of them in an easy way later.

The correction for overlap values were calculated by using the product of overlap with previously implemented measures, with a minimum value of 0.2

As a last, the marginal costs were calculated.

#### Making random values

In this part, a definition is written to put the values for the variables in the right order for each country. Then, random values are calculated for each of the variables.

Then, definitions are written to specify which RP values belong to which costs. This will be used later.

Reduction potentials and costs

In this part, definitions are written to calculate the cumulative reduction potentials and the costs. In these definitions the year and the country need to be specified.

#### Run a 1000 times

Definitions are written to calculate the list of RP values a 1000 times. These are still definitions and the country and year need to be specified. These definitions are used to calculate the list of RP values a 1000 times for each country and for the years 2020, 2050 and 2100. This is defined as step1_2020 etc.

Calculate the mean, 95th percentile and 5th percentile

The mean, 95th percentile and 5th percentile are calculated. Also, for each country and for each of these percentiles, the 2020, 2050 and 2100 values are put into one list. The values are divided by 100. Then, the time and x values are calculated. These are needed for the excel file.

#### Export to excel

Here the data is exported to an excel file. Here the differentiation is made between the different countries. For N2O fertilizer, some countries have the first option and some the second.

#### Plotting

Here graphs are made.
